# Immunization with Virus-Like Particles Displaying Ara h 2‑Derived Peptides to Mitigate Peanut Allergy

**DOI:** 10.1021/acsnanomed.6c00076

**Published:** 2026-06-20

**Authors:** Johanna M. Smeekens, Matthew C. Jenkins, Kahlia Carl, Jessica R. Humphrey, Andrew V. Turner, James W. Krempski, Soheila J. Maleki, Ananda R. Podilapu, Brian P. Vickery, M. G. Finn, Michael D. Kulis

**Affiliations:** † Department of Pediatrics, Division of Allergy and Immunology, School of Medicine, 6797 University of North Carolina, Chapel Hill, North Carolina 27599, United States; ‡ University of North Carolina Food Allergy Initiative, School of Medicine, University of North Carolina, Chapel Hill, North Carolina 27599, United States; § Curriculum in Toxicology and Environmental Medicine, School of Medicine, University of North Carolina, Chapel Hill, North Carolina 27599, United States; ∥ School of Chemistry and Biochemistry, 1372 Georgia Institute of Technology, Atlanta, Georgia 30332, United States; ⊥ Agricultural Research Service, Southern Regional Research Center, United States Department of Agriculture, New Orleans, Louisiana 70124, United States; # Department of Pediatrics, 12239 Emory University School of Medicine and Children’s Healthcare of Atlanta, Atlanta, Georgia 30322, United States; ∇ School of Biological Sciences, Georgia Institute of Technology, Atlanta, Georgia 30332, United States

**Keywords:** peanut allergy, food allergy, allergy immunotherapy, virus-like particle (VLP), encapsulin (Enc), Ara h 2

## Abstract

Peanut allergy is caused by aberrant IgE-driven immune responses to a well-defined set of peptide sequences within allergenic proteins. Oral and sublingual desensitization therapies are associated with the elicitation of IgG antibodies against allergenic proteins that interfere with IgE-induced effects. We tested whether inducing peptide-specific IgG responses by displaying key epitopes on immunogenic protein nanoparticle platforms protected against peanut allergy. A panel of 10 IgE epitopes from Ara h 2, the major degranulation-inducing peanut allergen, were displayed on two virus-like particles (VLPs): modified versions of both the bacteriophage PP7 coat protein and the iron-regulating encapsulin nanocompartment of the soil bacterium *Myxococcus xanthus*. Two peptide epitopes (Ah2-1, DPYSPS­QDPYSP­SPYDR­RGAGSSQ, and Ah2-6, HASAR­QQWEL­QGDRR­CQSQL­ERAN) were found to elicit strong and selective serum antibody responses against the full-length Ara h 2 protein. Immunization with a heterologous schedule (priming with PP7-displayed peptides, boosting with the corresponding encapsulin, and adjuvanting with the laboratory analogue of the clinical squalene-based adjuvant AS03) protected BALB/cJ mice against anaphylaxis in challenges with full-length Ara h 2 and whole peanut extract. These results suggest that focusing IgG-generating immune responses against specific peptide epitopes may be an effective prophylactic strategy against food allergies for which dominant epitopes are known.

## Graphical abstract

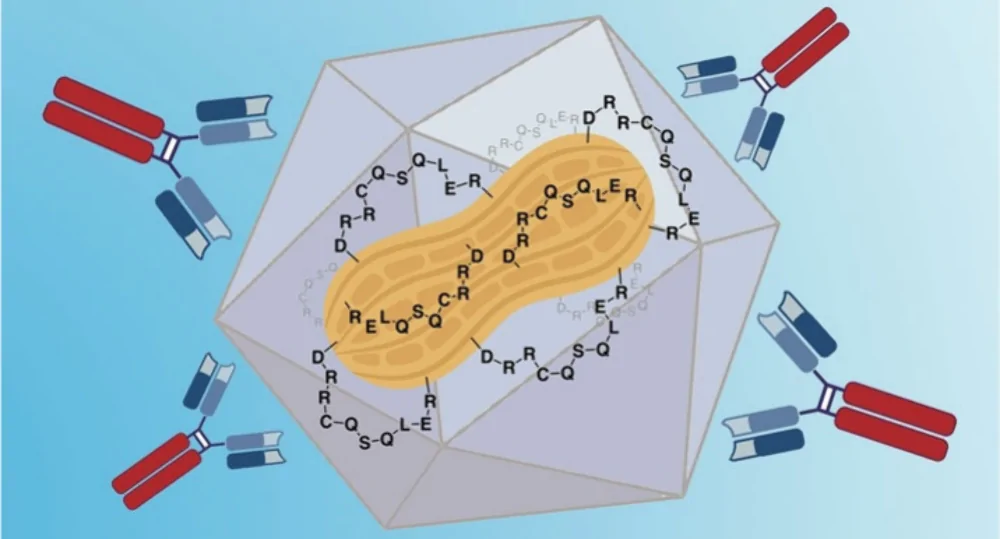

## Introduction

Food allergy is a potentially life-threatening disease that impacts an estimated 10% of the US population.^1^ Failure of oral tolerance mechanisms creates a Th2-skewed immune response to food antigens, leading to the production of IgE^2,3^ which binds to FcεRI on mast cells and basophils, priming these effector cells for degranulation upon subsequent allergen exposure. Inhibitory antibodies, primarily of the IgG isotype, can interfere with IgE-mediated degranulation by neutralizing allergens prior to binding IgE and/or by direct binding to FcγRIIb on effector cells causing inhibitory cell signaling.^4,5^ Therefore, induction of antigen-specific IgG, particularly IgG4, is a desired response for food allergy therapeutics and preventative approaches,^6–8^ with mechanistic studies demonstrating that IgG induced by oral and sublingual immunotherapy (OIT and SLIT, respectively) have inhibitory functions that prevent IgE-mediated degranulation.^5,9–16^ A disadvantage of OIT and SLIT is the requirement of daily dosing over many months or years; therefore therapeutic approaches that induce robust IgG responses without requiring daily dosing would be a major breakthrough. For environmental allergies, a clinical trial using monoclonal IgG4 antibodies specific to cat allergen reduced allergic symptoms *in vivo*, providing further evidence that IgG antibodies are effective allergy therapies.^17^ An ongoing clinical trial using monoclonal IgG4 antibodies for the major peanut allergen, Ara h 2, will evaluate this approach in food allergy.^18^

Virus-like particles (VLPs) are noninfectious, recombinantly produced biomolecules that resemble viruses in their physical structures and ability to engage the immune system.^19,20^ VLP scaffolds routinely elicit predominant IgG responses after subcutaneous or intramuscular injection, being well-suited to lymph node trafficking, dendritic cell uptake and processing to MHC presentation, and B and T cell cross-talk, leading to reliable class-switching and affinity maturation.^20–23^ Our group^24–26^ along with many others^22,27,28^ have developed VLPs into versatile platforms by adding peptide extensions or loops by genetic encoding^29–31^ or bioconjugation chemistry.^32,33^ These modifications allow for the construction of multifunctional VLP-based immunogens that display both immune cell receptor-targeting and antigen motifs.^34,35^ To focus the immune response on the desired peptide epitopes, we have immunized and boosted with different immunogenic particles that share only the desired peptide epitope.^36^

Here, we aimed to prevent peanut allergy in mice through prophylactic immunization with protein nanoparticle scaffolds presenting peptides from Ara h 2, the major peanut allergen responsible for eliciting degranulation,^37,38^ having well-defined IgE-binding epitopes across its relatively small 160 amino acid sequence.^39–43^ We generated peptides ranging in size from 14 to 41 amino acids with full coverage of the IgE-binding sites and genetically incorporated them into surface-accessible regions of engineered PP7 VLPs or particles derived from the *Myxococcus xanthus* encapsulin (Enc),^36^ to display the relevant peptides in conformationally restricted fashion. In addition to inducing an epitope-specific IgG response to inhibit allergen binding to IgE, we also employed a Th1-promoting adjuvant, squalene, during immunization with VLP/Enc, which was found to be required to prevent anaphylaxis during peanut exposure. Our peptide-based approach is complementary to the use of a full-length Ara h 2-VLP, currently being investigated in a Phase 1 clinical trial.^44,45^ Using particular peptide antigens allows us to focus on known immunodominant allergenic sequences, which may increase efficacy while providing a better safety profile because short peptides are less likely than full length allergens to cross-link IgE.^46^

## Results and Discussion

### Ara h 2 Peptide VLP and Enc Design and Characterization

We designed 20 VLP or Enc particles to display ten unique peptides from Ara h 2 that targeted six IgE epitopes (Table 1). Importantly, these human IgE binding epitopes are similar to mouse IgE epitopes, providing a translational platform to test the prevention of IgE-mediated anaphylaxis.^47^ Table 1 shows the entire sequence of the Ara h 2.01 isoform,^40^ with IgE epitopes highlighted in red, and gray boxes representing the amino acids included in the nanoparticle constructs. Candidate peptides were genetically incorporated into the protomer sequences of the two nanoparticle scaffolds as either linear C-terminal extensions, or as grafts within select solvent-exposed loops that have previously shown tolerance to peptide insertions (Figure 1, Figure S1A).^31,36^

**Figure 1:**
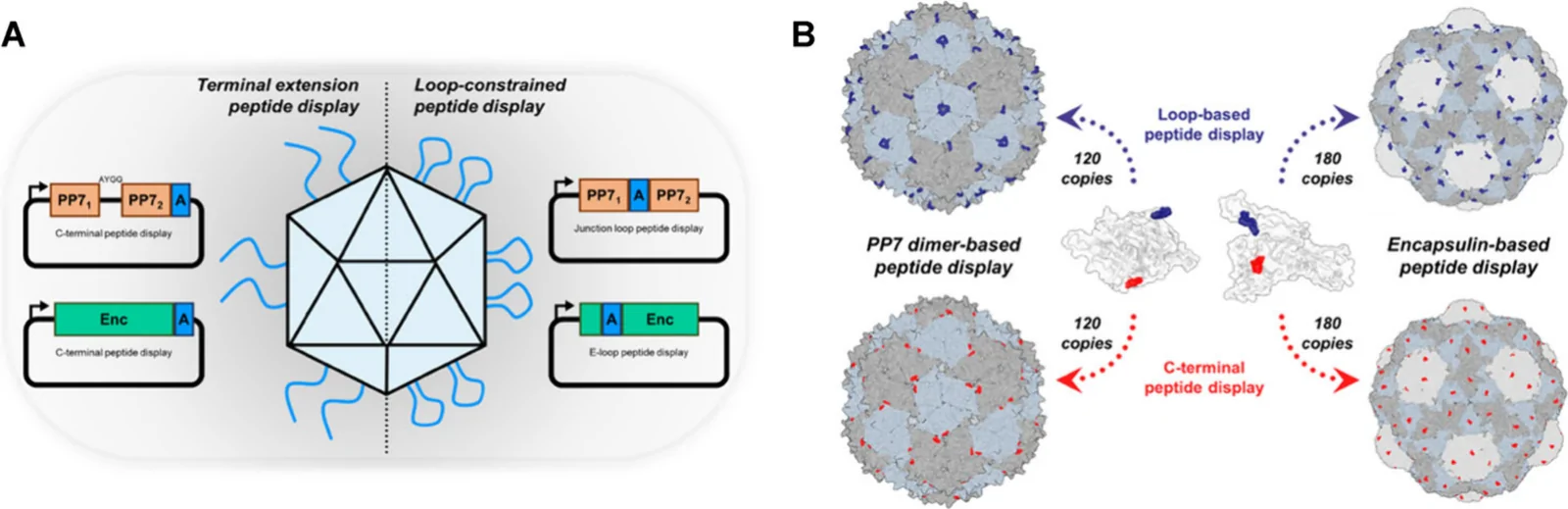
(A) Two methods of genetically encoding allergenic peptide epitopes into the protomer sequences of PP7 and *M. xanthus* encapsulin nanoparticles. (B) Structures of the two platforms used here, showing the placement of the peptide insertions; representative transmission electron microscopy images are shown in Figure S1B.

**Table 1:**
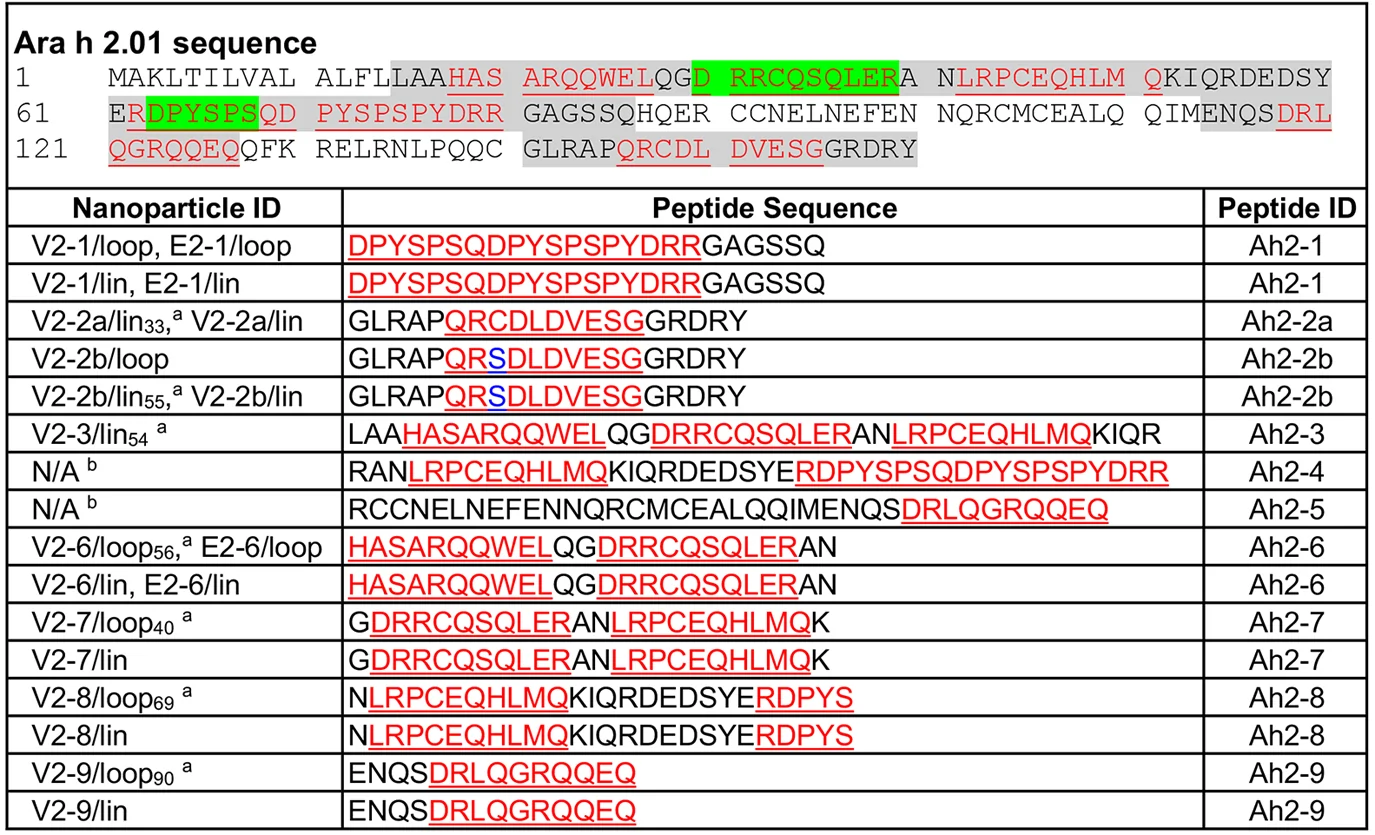
Ara h 2 Sequence with IgE Binding Epitopes Underlined in Red, the Two Immunodominant Sequences Highlighted in Green,^49,50^ VLP/Enc Peptide Coverage in Gray, with Corresponding VLP Peptide Sequences and Peptide IDs^a^

* Unless noted otherwise, “linear” and “loop” particles displayed 180 and 120 copies, respectively, of the indicated peptide sequence. Ara h 2 peptides 2a and 2b are distinguished by a substitution of the natural Cys residue with Ser (blue) to avoid nanoparticle aggregation upon multivalent display.

a Expressed as a hybrid particle with the wild-type PP7 sequence, subscript numbers represent the average number of peptides displayed per hybrid VLP.

b Omitted from this study because of an inability to produce assembled particles.

The VLP and Enc variants were recombinantly produced in *E. coli* and purified to homogeneity following previously established isolation protocols (Supporting Information). Encouragingly, most of the nanoparticle variants retained the capacity to assemble into complete protein cages with the foreign peptide sequences included (Figures S2–S4), thus yielding PP7- and Enc-based scaffolds with 120 or 180 displayed peptide copies, respectively. Of the eight designed PP7-peptide variants that failed to assemble during *in vivo* expression, five (VLPs V2-3/lin, V2-6/loop, V2-7/loop, V2-8/loop, and V2-9/loop) were subsequently isolated as fully assembled cages with reduced peptide valency (approximately 40–90 peptide copies per assembled VLP) by coexpressing the peptide-modified protomer gene alongside an unmodified protomer gene copy (Figure S5).^29^ Two variants (VLPs V2-2a/lin and V2-2b/lin) were initially produced as reduced valency hybrid VLPs, but were subsequently isolated as fully homogeneous VLPs with 120 peptides copies per cage after modifying *in vivo* expression conditions. The remaining three variants, all encoding either the Ah2-4 and Ah2-5 peptides, failed to form viable nanoparticles by either recombinant expression method. All successfully purified nanoparticles were characterized by dynamic light scattering and transmission electron microscopy to verify sample homogeneity and polydispersity prior to immunological tests (Figures S2–S4). We note that these particles can become more morphologically diverse upon peptide addition to loops, isolated as mixtures of closed structures of varying symmetry.^36,48^ These components are inseparable by our standard methods (sucrose gradient ultracentrifugation, size-exclusion chromatography) but morphological diversity does not appear to have a significant impact on immunogenicity.^36^ For convenience, we refer to these particles as their canonical icosahedral structures.

### Immunogenicity of Ara h 2 Peptide-Displaying Nucleoprotein Nanoparticles

Two epitopes within Ara h 2, DPYSPS and DRRCQSQLER, have consistently been identified across multiple laboratories as the immunodominant epitopes for human IgE in patients with confirmed peanut allergy.^16,41,43,49–53^ These epitopes are present in the Ah 2-1, 2-4 and Ah 2-3, 2-6 peptides, respectively (Table 1). To determine the immunogenicity of the VLP/Encs, we subcutaneously immunized BALB/cJ mice on days 1 and 21 with VLP/Encs, using the same peptide-displaying particle for both the prime and boost immunization (Figure 2A). No adjuvant was employed in these experiments, since VLPs of this type are known to be self-adjuvanting by virtue of TLR7/8 agonism from the bacterial RNA encapsidated during recombinant particle expression.^54,55^ Serum IgG responses assessed at day 36 showed complete peptide specificity against the Ah2-1 sequence (Figure 2B) and substantial specificity against Ah2-6, with some cross-reactivity toward Ah2-3 and Ah2-7, which share the DRRCQSQLER motif (Figure 2C). In both cases, immunization with either linear or loop presentations of the peptides produced IgG antibodies that bound immobilized linear peptide in the ELISA assay. Immunizations with other VLP-peptides were similarly selective for their respective epitopes (data not shown), but these proved to be of lesser interest in light of the observed cross-reactivity of serum antibodies with the more meaningful full-length Ara h 2 and peanut extract targets. The IgG assays (Figure 2D,E) showed strong Ara h 2- and peanut-specific IgG from immunizations with the Ah2-1 and Ah2-6 peptides, again for both linear and loop presentations, and low but detectable binding from Ah2-3- and Ah2-7 based immunogens. Similar low binding above background levels was also observed for serum from immunization with particles bearing Ah2-2; a curious result was the significantly better performance of the hybrid particle displaying only 33 copies of the linear peptide (V2-2a/lin_33_) relative to the construct with 120 such peptides (V2-2a/lin). We therefore did not include the Ah2-2 based particles in subsequent experiments.

**Figure 2:**
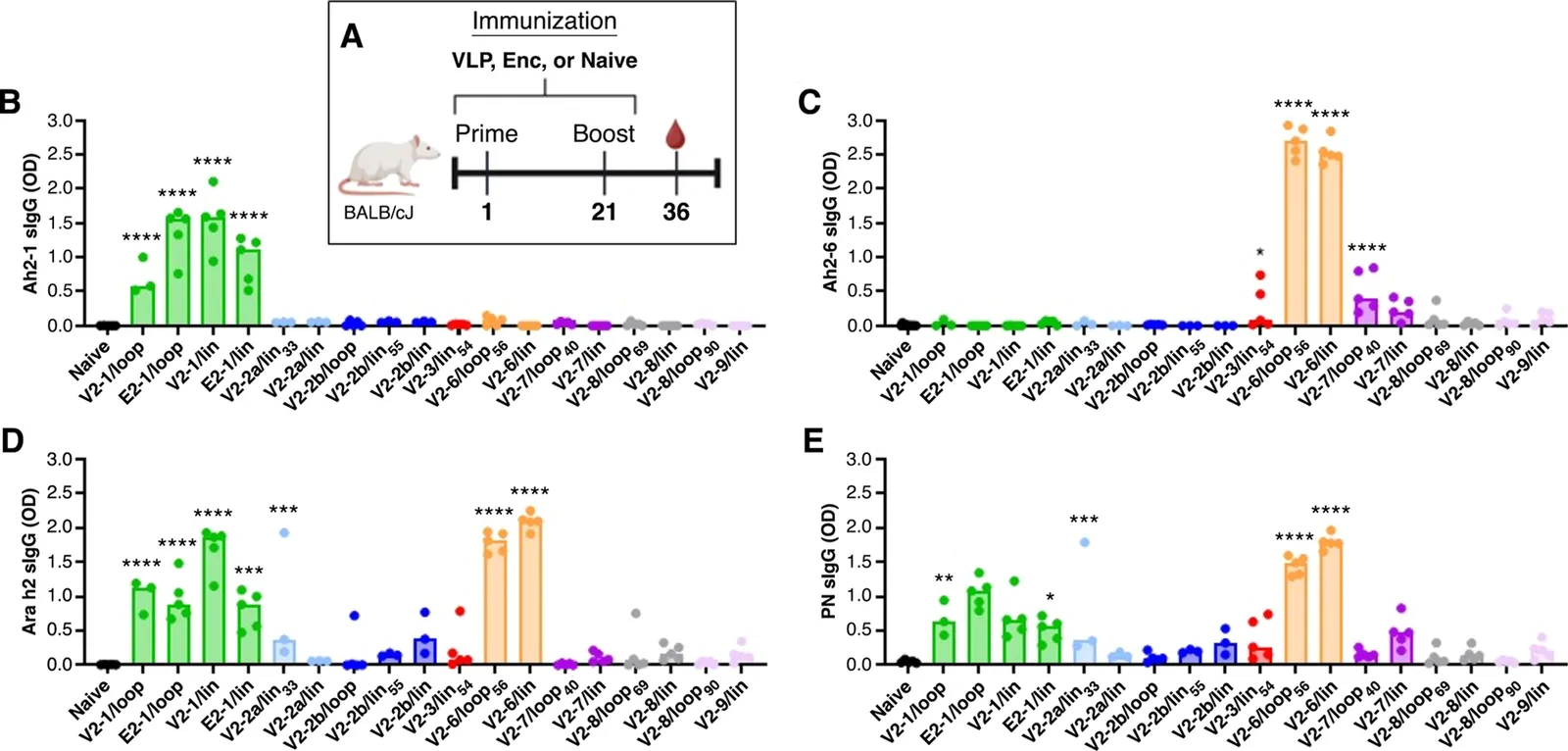
Serum IgG responses by ELISA (day 36) from immunized mice (panel A = immunization schedule) treated with the immunogen shown on the horizontal axis, against the following plated antigens: (B) linear Ah2-1 peptide, (C) linear Ah2-6 peptide, (D) full-length Ara h 2, and (E) peanut extract. Values are absorbance at 450 nm in the ELISA protocol (1:5000 serum dilution) as described in the Experimental Section. Individual data are shown with bars representing mean + SEM *p < 0.05, **p < 0.01, ***p < 0.001, ****p < 0.0001, one-way ANOVA, comparisons are between the naïve group and each immunization group.

Since only Ah2-1 and Ah2-6 based particles induced consistent and significant full-length Ara h 2-specific IgG, we selected them to test in a mouse model of peanut allergy, with the aim of inducing protective IgG responses and preventing anaphylaxis. Homogeneous prime-boost immunization with encapsulin displaying the Ah2-1 peptide was as effective as PP7 (Figure 2B, D, E). This set the stage for heterologous immunization, which we have adopted as a convenient method to minimize carrier epitope distraction.^36^ To take advantage of the same platform switching for the Ah2-6 epitope, we also generated the encapsulin E2-6/loop and E2-6/lin particles displaying the Ah2-6 peptide.

### Prevention of Anaphylaxis with Ara h 2 Peptide VLPs and Enc

To implement a well-established model of peanut allergy,^56,57^ we initially immunized BALB/cJ mice with PP7 particles V2–1/lin and V2–6/loop on day 1, and then boosted with encapsulin particles E2-1/loop, E2-1/lin, E2-6/loop, and E2-6/lin on day 21. Separate groups either received or lacked the clinically relevant squalene-based adjuvant AddaS03, included to test the effects of further enhancing the immune response in this manner.^58^ Beginning at day 36 after initial immunization, the mice underwent a peanut sensitization regimen by oral gavage with peanut and cholera toxin once weekly for 4 weeks, followed by an Ara h 2 challenge on day 65 and a peanut challenge 6 weeks later (Figure 3A). Because both IgE and IgG can mediate anaphylaxis in mice^59^ (whereas in humans, anaphylaxis to peanut is only caused by IgE), we blocked the IgG-mediated anaphylaxis pathway by injecting monoclonal antibodies against FcγRII (CD32) and FcγRIII (CD16) 24 h before challenge.^60^ This approach allows the circulating Ara h 2-specific IgG induced by immunization to still neutralize Ara h 2 allergen and protect from IgE-mediated anaphylaxis while minimizing any IgG-mediated degranulation.

**Figure 3:**
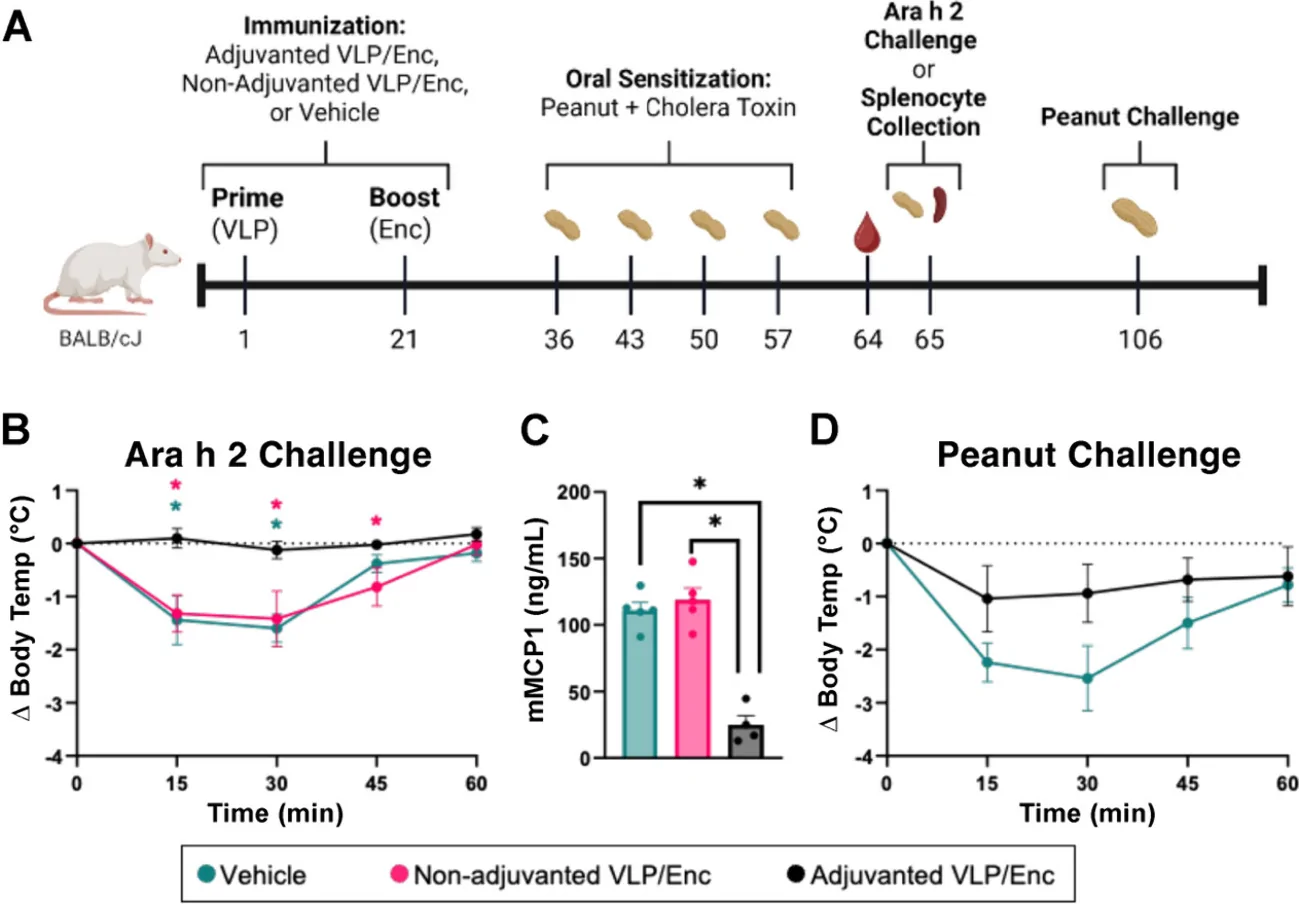
Immunization with VLPs and Encs to prevent peanut allergy. (A) Experimental schematic for prevention study, including immunization with VLP (prime) and Enc (boost), oral sensitization to peanut, blood collection, spleen harvest, Ara h 2 challenge, and peanut challenge. Anaphylaxis outcomes based on body temperature data from intraperitoneal challenge to (B) Ara h 2 with resulting (C) MMCP-1 release in serum, and (D) peanut protein extract. Challenge data and MMCP-1 are plotted as mean ± SEM *p < 0.05, Mann–Whitney U test, comparisons are between the adjuvanted VLP/Enc group and vehicle or nonadjuvanted VLP/Enc groups.

Mice immunized with adjuvanted VLP/Enc were completely protected from Ara h 2-induced anaphylaxis, as indicated by no change in body temperature upon challenge (Figure 3B), whereas the vehicle-treated mice and those receiving the vaccine without AddaS03 experienced the same degree of anaphylaxis, demonstrated by hypothermia. Mouse mast cell protease-1 (MMCP-1) quantified at the 60 min time point provided additional evidence that IgE-mediated anaphylaxis was inhibited in the mice immunized with adjuvanted VLP/Enc but not in the other treatment groups (Figure 3C). Furthermore, mice immunized with adjuvanted VLP/Enc were partially protected from anaphylaxis upon whole peanut challenge, with less severe body temperature decreases compared to vehicle-treated mice (Figure 3D). The extract used in this experiment was prepared from peanut flour that contains additional major peanut allergens including Ara h 1, Ara h 3, and Ara h 6,^43^ which likely caused the observed mild reaction. This suggests that VLP/Enc targeting of these other major allergens should be beneficial for protection against whole peanut challenge.

### Immune Modulation with Ara h 2 Peptide VLPs and Enc

Mice were bled on day 64 after the peanut sensitization period to measure the protective antibody response induced by Ara h 2 peptide VLP/Enc.^56^ The adjuvanted VLP/Enc treated mice had significantly increased Ara h 2-specific IgG, IgG2a and IgG1 responses compared to vehicle, consistent with the observed protection from Ara h 2-induced anaphylaxis (Figure 4A). Furthermore, these mice had significantly decreased Ara h 2-specific IgE, compared to vehicle. Increased allergen-specific IgG with concomitant decreased IgE has been previously reported in clinical trials of peanut OIT and SLIT.^61^ The nonadjuvanted VLP/Enc group had a more modest response in terms of IgG production and IgE reduction. Peptide-specific data were consistent with the above findings as well. Thus, mice immunized with adjuvanted VLP/Enc had significantly increased induction of IgG1, IgG2a, and total IgG against the linear Ara h 2 peptides, Ah2-1 and Ah2-6, compared to vehicle (Figure 4B, C). There was minimal IgE produced against Ah2-1 and Ah2-6 across all treatment groups, with quantities below the limit of quantification. These antibody responses are consistent with protection against anaphylaxis by IgG production and binding to Ah2-1, Ah2-6, and related epitopes on Ara h 2, masking the immunodominant IgE epitopes.

**Figure 4:**
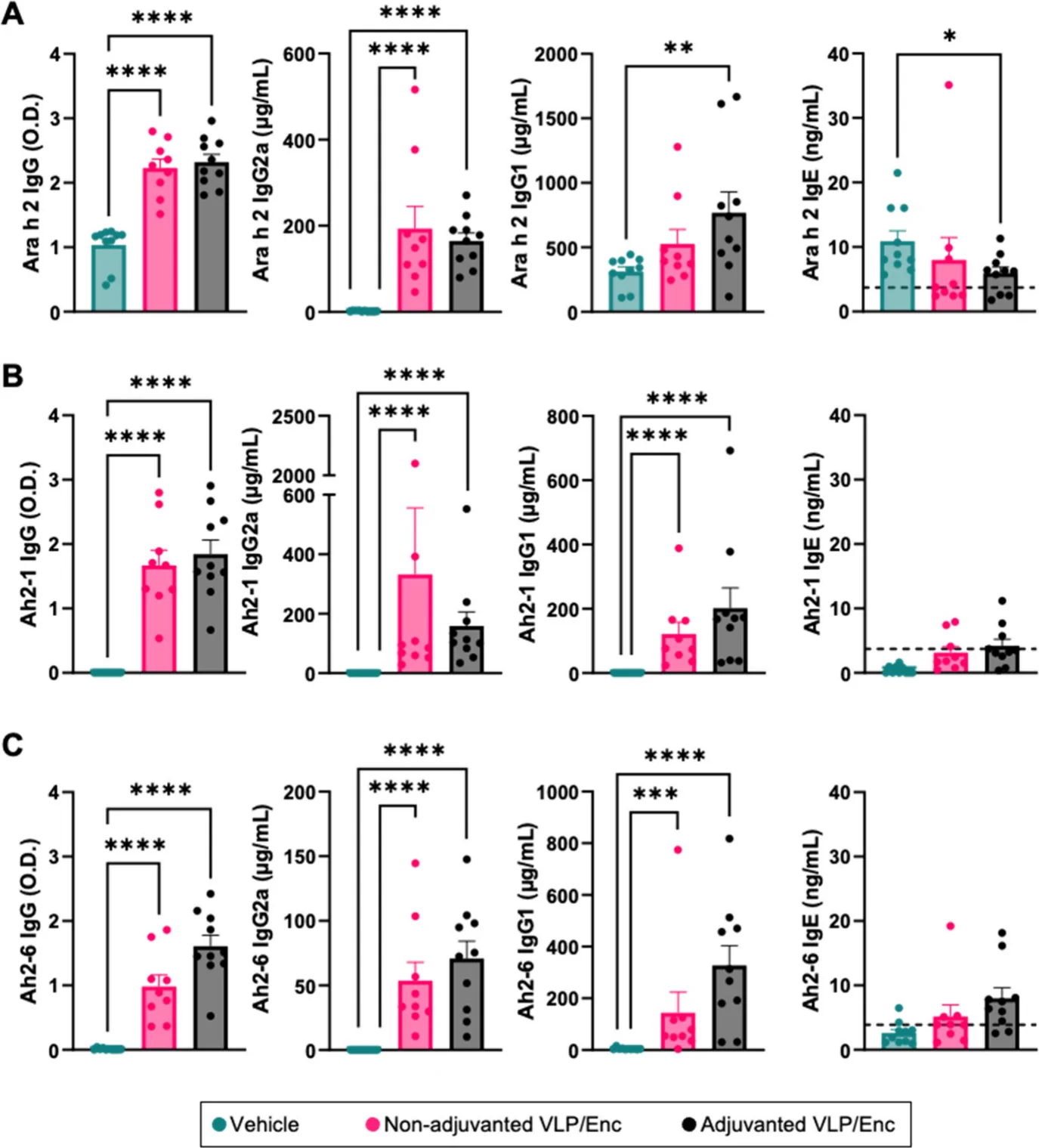
Antibody response after immunization with VLPs and Encs and peanut sensitization. (A) Ara h 2-specific IgG, IgG2a, IgG1, and IgE. (B) Ah2-1 peptide-specific IgG, IgG2a, IgG1, and IgE. (C) Ah2-6 peptide-specific IgG, IgG2a, IgG1, and IgE. Individual data are shown with bars representing mean + SEM; dashed horizontal line indicates limit of quantification. *p < 0.05, **p < 0.01, ***p < 0.001, ****p < 0.0001. Mann–Whitney U test, comparisons are between the vehicle group and each treatment group.

T cell modulation was measured by secreted cytokine production from Ara h 2- and peanut-stimulated splenocytes. Briefly, spleens were harvested on day 65 from mice that had undergone immunization and sensitization, but not an allergen challenge (Figure 3A). After culturing splenocytes in the presence of Ara h 2 or peanut extract for 96 h, the amounts of Th2, Th1 and Treg associated cytokines in the supernatants were measured. Overall, the Ara h 2-induced Th2 cytokines, IL-4, IL-13 and IL-9 were decreased in mice treated with VLP/Enc, with or without adjuvant, with statistically lower IL-4 in the adjuvanted VLP/Enc treated mice (Figure 5A). The Treg cytokine, IL-10, was also statistically lower in mice treated with adjuvanted VLP/Enc, compared to vehicle (Figure 5A). Similarly, the Th1 cytokines, IFNγ and TNFα were lower in the adjuvanted VLP/Enc group, whereas no change was observed in IL-2 between groups. Given the suppression in both Th2 and Th1 responses, it is possible that antigen-specific hyporesponsiveness was induced. For peanut-stimulated responses, the overall trends were similar to the Ara h 2-stimulated response, although there were no statistically significant differences, likely because peanut contains other allergens that were not modulated by the Ara h 2 peptide VLP/Enc (Figure 5B). The suppression of cytokine production in an Ara h 2-specific fashion, particularly the potent Th2 cytokine IL-4, has been shown to occur in peanut OIT and SLIT in humans,^62,63^ suggesting the therapeutic potential of this adjuvanted VLP/Enc approach.

**Figure 5:**
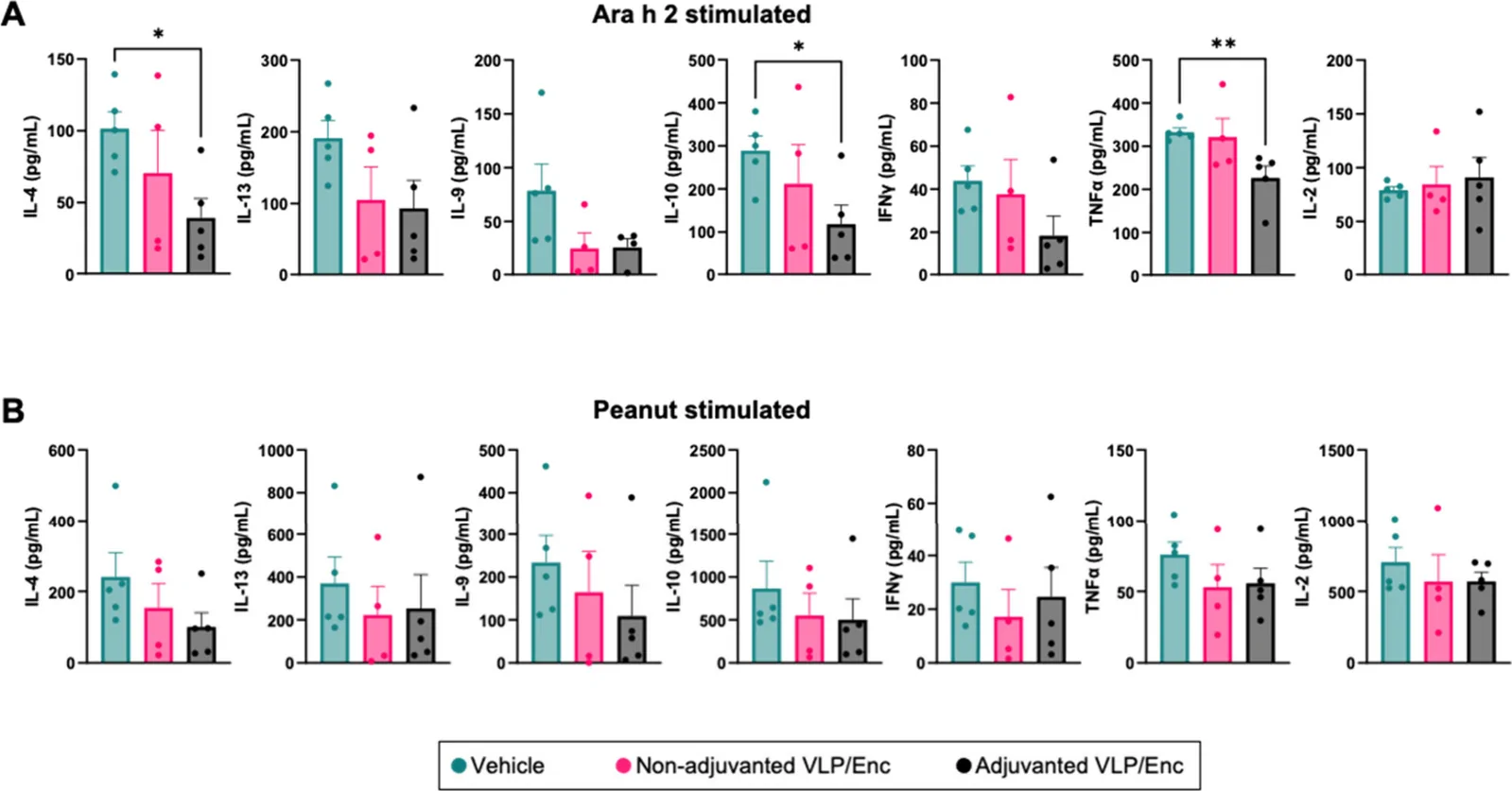
T cell cytokine responses after VLP and Enc immunization and peanut sensitization. Secreted cytokines from splenocytes stimulated with (A) Ara h 2 and (B) peanut. Individual data are shown with bars representing mean + SEM *p < 0.05, **p < 0.01. Mann–Whitney U test, comparisons are between the vehicle group and each treatment group.

## Conclusion

The multivalent display of Ara h 2 peptides on virus-like and encapsulin protein nanoparticles was tested as an approach for preventing peanut allergy, with potential clinical implications of reducing the amount and frequency of antigen exposure required to induce tolerance. After initial immunization experiments with VLP and Enc candidates, we identified two epitopes that were critical for Ara h 2-specific IgG production, DPYSP­SQDPY­SPSPYD­RRGAG­SSQ, and HASA­RQQW­ELQGD­RRCQS­QLERAN. These epitopes are also known as the most allergenic Ara h 2 motifs in humans. An optimized protocol for eliciting peptide-specific responses, involving the use of immunologically orthogonal platforms and the clinically relevant adjuvant AddaS03, provided complete protection from Ara h 2-induced anaphylaxis in a mouse model of induced allergy, consistent with the production of Ara h 2-specific IgG, IgG2a and IgG1. These data suggest that VLPs and Encs targeting only two Ara h 2 epitopes have potential as a therapeutic approach in mice with an established peanut allergy, presumably by activating IgG^+^ memory B cells and increasing effector cell thresholds.^64^ Indeed, human IgG4 antibodies raised against Ara h 2 epitopes have been shown to neutralize IgE-mediated degranulation,^16^ have preclinical efficacy in animal and human cellular systems,^65^ and are being studied in a Phase 1 clinical trial (NCT06331728). Although highly promising, this approach will rely on regular, repeated injections of IgG4 antibodies that will not modulate the underlying Th2 and IgE responses to peanut. The detailed mechanism of action of IgG molecules in this context is not certain, as they can mask antigen from IgE engagement or B cell stimulation and/or suppress IgE-effector pathways, among other roles.

While strong IgG responses were induced by Ara h 2 peptide-displaying nanoparticles, the results also suggest that IgG may be necessary but not sufficient. Nonadjuvanted and adjuvanted formulations gave similar antibody titers but only the latter proved effective against allergen-induced anaphylaxis. Importantly, we have yet to investigate epitope-IgG affinity and IgG neutralizing capacity, which may play a role in anaphylaxis protection,^16,41^ nor have we yet tested efficacy in a treatment model (such as with presensitized mice). Overall, the modular nature of nanoparticle-peptide display provides a clear path for extension of this approach to the other major peanut allergens as well as allergens from any food source to mitigate IgE-mediated food allergy.

## Experimental Section

### Reagents

Peanut protein extract was prepared from roasted, defatted peanut flour (Golden Peanut, Alpharetta, GA) in PBS supplemented with 1 M NaCl, as described previously.^66^ The peanut extract yielded a 21 mg/mL concentration by BCA assay, with individual allergen concentrations determined by commercially available ELISA kits from InBio (Charlottesville, VA) as follows: 0.68 mg/mL Ara h 1, 5.67 mg/mL Ara h 2, 13.35 mg/mL Ara h 3, 2.56 mg/mL Ara h 6. Ara h 2 was purified as previously described.^67,68^ Biotinylated peptides for immunological analysis were synthesized by standard Fmoc-based solid-phase methods; details are provided in Supporting Information.

### VLP and Enc Formulation and Characterization

Peanut peptides were genetically inserted into either dimeric PP7 or *M. xanthus* encapsulin genes using standard methods. Complete details are given in Supporting Information.

### Mice

Female BALB/cJ mice were obtained from Jackson Laboratories (Bar Harbor, ME) at 4 weeks of age. Mice were maintained under specific pathogen-free conditions, kept on a 12:12 light:dark cycle, and raised on standard mouse chow, free of peanut. All animal experiments were approved by the Institutional Animal Care and Use Committee at the University of North Carolina at Chapel Hill.

### Immunogenicity Assessment

Mice were treated on day 1 and 21 with 50 μg VLPs or Encs (listed in Table 1) by subcutaneous injection, or kept naïve. Mice were bled on day 36, and serum was isolated for antibody quantification.

### Prevention of Anaphylaxis

Mice were immunized with 50 μg V2-1/lin and V2-6/loop by subcutaneous injection on day 1 and immunized with 50 μg E2-1/loop, E2-1/lin, E2-6/loop, and E2-6/lin by subcutaneous injection on day 21, with or without the squalene adjuvant, AddaS03 (InvivoGen, San Diego, CA). On days 36, 43, and 50, mice were sensitized to peanut by oral gavage with 2 mg peanut extract and 10 μg cholera toxin (List Laboratories, Campbell, CA). On day 57, mice were sensitized to peanut by oral gavage with 5 mg peanut extract and 10 μg cholera toxin. Mice were bled on day 64 and serum was isolated to quantify immunoglobulins. The same day, mice were intraperitoneally injected with 2.4G2 (Bioxcell, Lebanon, NH), the monoclonal blocking antibody against FcγRII (CD32) and FcγRIII (CD16), to block IgG-mediated anaphylaxis. Then, 24 h later, on day 65, half of the mice from each group (n = 5) were challenged to 200 μg Ara h 2 by intraperitoneal injection. Core body temperatures were recorded every 15 min for 1 h postchallenge using a rectal probe (Physitemp, Clifton, NJ). At the 1 h time point, blood was collected to quantify MMCP-1 in serum using an ELISA kit (ThermoFisher, Waltham, MA). About 6 weeks later, on day 106, mice were challenged to 1 mg peanut extract by intraperitoneal injection, and body temperatures were recorded as described above. The other half of the mice from each group (n = 5) were sacrificed on day 65 and spleens were harvested for T cell cytokine analysis.

### T Cell Cytokine Analysis

Splenocytes were plated at a density of 5.0 × 10^6^ cells/mL in complete media (RPMI with 10% FBS, 100 U/mL penicillin, 100 μg/mL streptomycin, and 2 mM glutamine) and cultured with 200 μg/mL peanut extract or 100 μg/mL purified Ara h 2 for 96 h in a 37 °C, 5% CO_2_ humidified incubator. The secreted cytokines, IL-4, IL-13, IL-9, IL-10, IFNγ, TNFα, and IL-2, were quantified in supernatants by multiplex MSD assays (Meso Scale Discovery, Rockville, MD).

### Antigen-Specific IgG, IgG2a, IgG1, and IgE ELISAs

Peanut-specific, Ara h 2-specific, Ah2-1 peptide-specific, and Ah2-6 peptide-specific IgG, IgG2a, IgG1 and IgE were quantified from serum samples by ELISA. For peanut- and Ara h 2-specific ELISAs, 96-well plates were coated with 20 μg/mL peanut protein extract or 5 μg/mL purified Ara h 2 for samples, and 20 μg/mL HSA-DNP (Millipore Sigma, St. Louis, MO) for standard curves (IgG2a, IgG1, IgE plates). Plates were blocked with 2% BSA in PBS-0.05% Tween. For peptide-specific IgG, IgG2a, IgG1, and IgE ELISAs, 96-well plates were coated with 1 μg/mL streptavidin (ThermoFisher, Waltham, MA) for samples, and 20 μg/mL HSA-DNP (Millipore Sigma) for standard curves (IgG2a, IgG1, IgE plates). Plates were blocked with 0.5% BSA in PBS-0.05% Tween. Biotinylated Ah2-1 and Ah2-6 peptides were diluted in blocking buffer and added to plates at a concentration of 10 μg/mL. Serum samples were diluted 1:20 for IgE, 1:20,000 for IgG1, 1:1,000 for IgG2a, and 1:5,000 for IgG. Standard curves were prepared by serial dilution for mouse IgG2a (0.002–2 μg/mL, Accurate Chemicals, Westbury, NY), IgG1 (0.002–2 μg/mL, Accurate Chemicals), and IgE (0.06–62.5 ng/mL, Accurate Chemicals). Detection antibodies for each isotype were: goat-anti mouse IgG­(H+L)-HRP (1:2,000, Invitrogen, Carlsbad, CA), goat antimouse IgG2a-HRP (1:20,000, Southern Biotech, Birmingham, AL), goat antimouse IgG1-HRP (1:40,000, Southern Biotech), or goat antimouse IgE-HRP (1:5,000, Southern Biotech). All plates were developed using TMB (SeraCare, Milford, MA) and quenched using 1% HCl (SeraCare). All plates were read on a plate spectrophotometer (BioTek, Winooski, VT) at 450 nm. Antigen-specific IgG2a, IgG1 and IgE concentrations were calculated based on the standard curve and dilution factor. Antigen-specific IgG is presented as O.D. values.

## Supplementary Material



## Notes

DNA sequences of primers and protein nanoparticle constructs; nanoparticle characterization data (PDF)
