# Supplementary material for: Immunization with Virus-Like Particles Displaying Ara h 2‑Derived Peptides to Mitigate Peanut Allergy
Source: ACS Nano Med. 2026 Jun 20;1(8):1987–96. doi: 10.1021/acsnanomed.6c00076 (PMC13564887; doi:10.1021/acsnanomed.6c00076)
Supplement: Supplementary file 1 [file nm6c00076_si_001.pdf]

Supporting Information

Immunization with virus-like particles displaying Ara h 2-derived peptides to mitigate peanut allergy

Johanna M. Smeekens[1,2,3] *, Matthew C. Jenkins[4], Kahlia Carl[4], Jessica R. Humphrey[1,2,3], Andrew V. Turner[1,2], James W. Krempski[1,2], Soheila J. Maleki[5], Ananda R. Podilapu[4], Brian P. Vickery[6], M.G. Finn[4,7] *, Michael D. Kulis[1,2]

[1] Department of Pediatrics, Division of Allergy and Immunology, School of Medicine, University of North Carolina, Chapel Hill, NC, USA 27599

[2] University of North Carolina Food Allergy Initiative, School of Medicine, University of North Carolina, Chapel Hill, NC, USA 27599

[3] Curriculum in Toxicology and Environmental Medicine, School of Medicine, University of North Carolina, Chapel Hill, NC, USA 27599

[4] School of Chemistry and Biochemistry, Georgia Institute of Technology, Atlanta, GA, USA 30332

[5] United States Department of Agriculture, Agricultural Research Service, Southern Regional Research Center, New Orleans, LA, USA 70124

[6] Department of Pediatrics, Emory University School of Medicine and Children's Healthcare of Atlanta, Atlanta, GA, USA 30322

[7] School of Biological Sciences, Georgia Institute of Technology, Atlanta, GA, USA 30332

*Corresponding authors

J.M. Smeekens
116 Manning Dr.
Mary Ellen Jones, Room 3310
Chapel Hill, NC 27599
johanna_smeekens@med.unc.edu

M.G. Finn
901 Atlantic Dr.
Atlanta, GA 30306
mgfinn@gatech.edu

I. DNA sequences of peanut epitope nanoparticles

a. Dimeric PP7-based peanut epitope nanoparticles

Upstream PP7 gene | Downstream PP7 gene | AYGG junction linker peptide
C-terminal 8 amino acid spacer | Ara h 2 peptide sequence
Start and stop codons are underlined and **bolded**, respectively.

V2-1/lin (C-terminal Ah2-1 peptide)

ATGAGCAAAACCATTGTTCTGAGCGTGGGTGAAGCGACCCGTACCCTGACCGAAATCCAGAGCACCGCTGACCGTCAAATTTTTGAGG
AAAAAGTGGGTCCGCTGGTTGGCCGTCTGCGTCTGACCGCGAGCCTGCGTCAGAACGGTGCGAAGACCGCGTACCGTGTGAACCTGA
AACTGGACCAAGCGGATGTGGTTGATTGCAGCACCAGCGTTTGCGGCGAGCTGCCGAAAGTGCGTTACACCCAGGTTTGGAGCCACG
ATGTGACCATCGTTGCGAACAGCACCGAAGCGAGCCGTAAGAGCCTGTATGATCTGACCAAAAGCCTGGTGGCGACCAGCCAAGTTGA
GGACCTGGTGGTTAATCTTGTACCACTTGGTCGCGCATATGGCGGTTCGAAAACCATCGTCCTGTCCGTGGGCGAAGCAACCCGCACC
CTGACCGAAATCCAATCTACCGCAGACCGCCAAATCTTTGAAGAAAAAGTGGGTCCGCTGGTCGGTCGTCTGCGTCTGACCGCCTCTC
TGCGTCAGAACGGCGCGAAAACGGCCTATCGCGTCAATCTGAAACTGGATCAAGCAGACGTGGTTGATTGCAGCACCTCTGTTTGTGG
TGAACTGCCGAAAGTGCGTTATACGCAGGTTTGGTCACATGACGTCACCATTGTGGCAAACTCGACGGAAGCTAGTCGCAAATCCCTGT
ACGATCTGACCAAATCCCTGGTGGCGACCTCTCAAGTGGAAGACCTGGTGGTGAACCTGGTGCCGCTGGGCCGCGGTGGCGCTAGCG
AAAGCGGCGCCGATCCGTATAGCCCATCTCAGGACCCATACTCGCCGAGCCCTTACGACCGTCGCGGCGCTGGTTCGTCCCAA**TAA**

V2-2a/lin (C-terminal Ah2-2a peptide)

ATGAGCAAAACCATTGTTCTGAGCGTGGGTGAAGCGACCCGTACCCTGACCGAAATCCAGAGCACCGCTGACCGTCAAATTTTTGAGG
AAAAAGTGGGTCCGCTGGTTGGCCGTCTGCGTCTGACCGCGAGCCTGCGTCAGAACGGTGCGAAGACCGCGTACCGTGTGAACCTGA
AACTGGACCAAGCGGATGTGGTTGATTGCAGCACCAGCGTTTGCGGCGAGCTGCCGAAAGTGCGTTACACCCAGGTTTGGAGCCACG
ATGTGACCATCGTTGCGAACAGCACCGAAGCGAGCCGTAAGAGCCTGTATGATCTGACCAAAAGCCTGGTGGCGACCAGCCAAGTTGA
GGACCTGGTGGTTAATCTTGTACCACTTGGTCGCGCATATGGCGGTTCGAAAACCATCGTCCTGTCCGTGGGCGAAGCAACCCGCACC
CTGACCGAAATCCAATCTACCGCAGACCGCCAAATCTTTGAAGAAAAAGTGGGTCCGCTGGTCGGTCGTCTGCGTCTGACCGCCTCTC
TGCGTCAGAACGGCGCGAAAACGGCCTATCGCGTCAATCTGAAACTGGATCAAGCAGACGTGGTTGATTGCAGCACCTCTGTTTGTGG
TGAACTGCCGAAAGTGCGTTATACGCAGGTTTGGTCACATGACGTCACCATTGTGGCAAACTCGACGGAAGCTAGTCGCAAATCCCTGT
ACGATCTGACCAAATCCCTGGTGGCGACCTCTCAAGTGGAAGACCTGGTGGTGAACCTGGTGCCGCTGGGCCGCGGTGGCGCTAGCG
AAAGCGGCGCCGGGTTGCGTGCGCCTCAACGTTGTGACCTTGACGTAGAGAGCGGGGGGCGCGACCGCTAC**TAA**

V2-2b/lin (C-terminal Ah2-2b peptide)

ATGAGCAAAACCATTGTTCTGAGCGTGGGTGAAGCGACCCGTACCCTGACCGAAATCCAGAGCACCGCTGACCGTCAAATTTTTGAGG
AAAAAGTGGGTCCGCTGGTTGGCCGTCTGCGTCTGACCGCGAGCCTGCGTCAGAACGGTGCGAAGACCGCGTACCGTGTGAACCTGA
AACTGGACCAAGCGGATGTGGTTGATTGCAGCACCAGCGTTTGCGGCGAGCTGCCGAAAGTGCGTTACACCCAGGTTTGGAGCCACG
ATGTGACCATCGTTGCGAACAGCACCGAAGCGAGCCGTAAGAGCCTGTATGATCTGACCAAAAGCCTGGTGGCGACCAGCCAAGTTGA
GGACCTGGTGGTTAATCTTGTACCACTTGGTCGCGCATATGGCGGTTCGAAAACCATCGTCCTGTCCGTGGGCGAAGCAACCCGCACC
CTGACCGAAATCCAATCTACCGCAGACCGCCAAATCTTTGAAGAAAAAGTGGGTCCGCTGGTCGGTCGTCTGCGTCTGACCGCCTCTC
TGCGTCAGAACGGCGCGAAAACGGCCTATCGCGTCAATCTGAAACTGGATCAAGCAGACGTGGTTGATTGCAGCACCTCTGTTTGTGG
TGAACTGCCGAAAGTGCGTTATACGCAGGTTTGGTCACATGACGTCACCATTGTGGCAAACTCGACGGAAGCTAGTCGCAAATCCCTGT
ACGATCTGACCAAATCCCTGGTGGCGACCTCTCAAGTGGAAGACCTGGTGGTGAACCTGGTGCCGCTGGGCCGCGGTGGCGCTAGCG
AAAGCGGCGCCGGGTTGCGTGCGCCTCAACGTTCAGACCTTGACGTAGAGAGCGGGGGGCGCGACCGCTAC**TAA**

V2-3/lin (C-terminal Ah2-3 peptide)

ATGAGCAAAACCATTGTTCTGAGCGTGGGTGAAGCGACCCGTACCCTGACCGAAATCCAGAGCACCGCTGACCGTCAAATTTTTGAGG
AAAAAGTGGGTCCGCTGGTTGGCCGTCTGCGTCTGACCGCGAGCCTGCGTCAGAACGGTGCGAAGACCGCGTACCGTGTGAACCTGA
AACTGGACCAAGCGGATGTGGTTGATTGCAGCACCAGCGTTTGCGGCGAGCTGCCGAAAGTGCGTTACACCCAGGTTTGGAGCCACG
ATGTGACCATCGTTGCGAACAGCACCGAAGCGAGCCGTAAGAGCCTGTATGATCTGACCAAAAGCCTGGTGGCGACCAGCCAAGTTGA
GGACCTGGTGGTTAATCTTGTACCACTTGGTCGCGCATATGGCGGTTCGAAAACCATCGTCCTGTCCGTGGGCGAAGCAACCCGCACC
CTGACCGAAATCCAATCTACCGCAGACCGCCAAATCTTTGAAGAAAAAGTGGGTCCGCTGGTCGGTCGTCTGCGTCTGACCGCCTCTC
TGCGTCAGAACGGCGCGAAAACGGCCTATCGCGTCAATCTGAAACTGGATCAAGCAGACGTGGTTGATTGCAGCACCTCTGTTTGTGG
TGAACTGCCGAAAGTGCGTTATACGCAGGTTTGGTCACATGACGTCACCATTGTGGCAAACTCGACGGAAGCTAGTCGCAAATCCCTGT
ACGATCTGACCAAATCCCTGGTGGCGACCTCTCAAGTGGAAGACCTGGTGGTGAACCTGGTGCCGCTGGGCCGCGGTGGCGCTAGCG
AAAGCGGCGCCCTGGCAGCCCATGCGTCGGCGCGCCAGCAATGGGAATTACAAGGTGACCGCCGTTGTCAGTCACAGTTAGAGCGTG
CGAATTTGCGCCCCTGCGAGCAGCATCTTATGCAGAAGATTCAGCGT**TAA**

V2-6/lin (C-terminal Ah2-6 peptide)

ATGAGCAAAACCATTGTTCTGAGCGTGGGTGAAGCGACCCGTACCCTGACCGAAATCCAGAGCACCGCTGACCGTCAAATTTTTGAGG
AAAAAGTGGGTCCGCTGGTTGGCCGTCTGCGTCTGACCGCGAGCCTGCGTCAGAACGGTGCGAAGACCGCGTACCGTGTGAACCTGA
AACTGGACCAAGCGGATGTGGTTGATTGCAGCACCAGCGTTTGCGGCGAGCTGCCGAAAGTGCGTTACACCCAGGTTTGGAGCCACG
ATGTGACCATCGTTGCGAACAGCACCGAAGCGAGCCGTAAGAGCCTGTATGATCTGACCAAAAGCCTGGTGGCGACCAGCCAAGTTGA
GGACCTGGTGGTTAATCTTGTACCACTTGGTCGCGCATATGGCGGTTCGAAAACCATCGTCCTGTCCGTGGGCGAAGCAACCCGCACC
CTGACCGAAATCCAATCTACCGCAGACCGCCAAATCTTTGAAGAAAAAGTGGGTCCGCTGGTCGGTCGTCTGCGTCTGACCGCCTCTC
TGCGTCAGAACGGCGCGAAAACGGCCTATCGCGTCAATCTGAAACTGGATCAAGCAGACGTGGTTGATTGCAGCACCTCTGTTTGTGG
TGAACTGCCGAAAGTGCGTTATACGCAGGTTTGGTCACATGACGTCACCATTGTGGCAAACTCGACGGAAGCTAGTCGCAAATCCCTGT
ACGATCTGACCAAATCCCTGGTGGCGACCTCTCAAGTGGAAGACCTGGTGGTGAACCTGGTGCCGCTGGGCCGCGGTGGCGCTAGCG
AAAGCGGCGCCCACGCATCAGCTCGTCAACAGTGGGAGCTTCAGGGTGATCGTCGCTGTCAAAGTCAGTTGGAGCGTGCGAAT**TAA**

V2-7/lin (C-terminal Ah2-7 peptide)

ATGAGCAAAACCATTGTTCTGAGCGTGGGTGAAGCGACCCGTACCCTGACCGAAATCCAGAGCACCGCTGACCGTCAAATTTTTGAGG
AAAAAGTGGGTCCGCTGGTTGGCCGTCTGCGTCTGACCGCGAGCCTGCGTCAGAACGGTGCGAAGACCGCGTACCGTGTGAACCTGA
AACTGGACCAAGCGGATGTGGTTGATTGCAGCACCAGCGTTTGCGGCGAGCTGCCGAAAGTGCGTTACACCCAGGTTTGGAGCCACG
ATGTGACCATCGTTGCGAACAGCACCGAAGCGAGCCGTAAGAGCCTGTATGATCTGACCAAAAGCCTGGTGGCGACCAGCCAAGTTGA
GGACCTGGTGGTTAATCTTGTACCACTTGGTCGCGCATATGGCGGTTCGAAAACCATCGTCCTGTCCGTGGGCGAAGCAACCCGCACC
CTGACCGAAATCCAATCTACCGCAGACCGCCAAATCTTTGAAGAAAAAGTGGGTCCGCTGGTCGGTCGTCTGCGTCTGACCGCCTCTC
TGCGTCAGAACGGCGCGAAAACGGCCTATCGCGTCAATCTGAAACTGGATCAAGCAGACGTGGTTGATTGCAGCACCTCTGTTTGTGG
TGAACTGCCGAAAGTGCGTTATACGCAGGTTTGGTCACATGACGTCACCATTGTGGCAAACTCGACGGAAGCTAGTCGCAAATCCCTGT
ACGATCTGACCAAATCCCTGGTGGCGACCTCTCAAGTGGAAGACCTGGTGGTGAACCTGGTGCCGCTGGGCCGCGGTGGCGCTAGCG
AAAGCGGCGCCGGTGATCGTCGTTGCCAGAGCCAACTGGAACGTGCCAATCTTCGCCCATGCGAACAACATTTAATGCAAAAA**TAA**

V2-8/lin (C-terminal Ah2-8 peptide)

ATGAGCAAAACCATTGTTCTGAGCGTGGGTGAAGCGACCCGTACCCTGACCGAAATCCAGAGCACCGCTGACCGTCAAATTTTTGAGG
AAAAAGTGGGTCCGCTGGTTGGCCGTCTGCGTCTGACCGCGAGCCTGCGTCAGAACGGTGCGAAGACCGCGTACCGTGTGAACCTGA
AACTGGACCAAGCGGATGTGGTTGATTGCAGCACCAGCGTTTGCGGCGAGCTGCCGAAAGTGCGTTACACCCAGGTTTGGAGCCACG
ATGTGACCATCGTTGCGAACAGCACCGAAGCGAGCCGTAAGAGCCTGTATGATCTGACCAAAAGCCTGGTGGCGACCAGCCAAGTTGA
GGACCTGGTGGTTAATCTTGTACCACTTGGTCGCGCATATGGCGGTTCGAAAACCATCGTCCTGTCCGTGGGCGAAGCAACCCGCACC
CTGACCGAAATCCAATCTACCGCAGACCGCCAAATCTTTGAAGAAAAAGTGGGTCCGCTGGTCGGTCGTCTGCGTCTGACCGCCTCTC
TGCGTCAGAACGGCGCGAAAACGGCCTATCGCGTCAATCTGAAACTGGATCAAGCAGACGTGGTTGATTGCAGCACCTCTGTTTGTGG
TGAACTGCCGAAAGTGCGTTATACGCAGGTTTGGTCACATGACGTCACCATTGTGGCAAACTCGACGGAAGCTAGTCGCAAATCCCTGT
ACGATCTGACCAAATCCCTGGTGGCGACCTCTCAAGTGGAAGACCTGGTGGTGAACCTGGTGCCGCTGGGCCGCGGTGGCGCTAGCG
AAAGCGGCGCCAACTTACGTCCGTGTGAACAGCATCTTATGCAAAAGATCCAGCGCGACGAAGACTCCTATGAACGCGACCCATACTC
G**TAA**

V2-9/lin (C-terminal Ah2-9 peptide)

ATGAGCAAAACCATTGTTCTGAGCGTGGGTGAAGCGACCCGTACCCTGACCGAAATCCAGAGCACCGCTGACCGTCAAATTTTTGAGG
AAAAAGTGGGTCCGCTGGTTGGCCGTCTGCGTCTGACCGCGAGCCTGCGTCAGAACGGTGCGAAGACCGCGTACCGTGTGAACCTGA
AACTGGACCAAGCGGATGTGGTTGATTGCAGCACCAGCGTTTGCGGCGAGCTGCCGAAAGTGCGTTACACCCAGGTTTGGAGCCACG
ATGTGACCATCGTTGCGAACAGCACCGAAGCGAGCCGTAAGAGCCTGTATGATCTGACCAAAAGCCTGGTGGCGACCAGCCAAGTTGA
GGACCTGGTGGTTAATCTTGTACCACTTGGTCGCGCATATGGCGGTTCGAAAACCATCGTCCTGTCCGTGGGCGAAGCAACCCGCACC
CTGACCGAAATCCAATCTACCGCAGACCGCCAAATCTTTGAAGAAAAAGTGGGTCCGCTGGTCGGTCGTCTGCGTCTGACCGCCTCTC
TGCGTCAGAACGGCGCGAAAACGGCCTATCGCGTCAATCTGAAACTGGATCAAGCAGACGTGGTTGATTGCAGCACCTCTGTTTGTGG
TGAACTGCCGAAAGTGCGTTATACGCAGGTTTGGTCACATGACGTCACCATTGTGGCAAACTCGACGGAAGCTAGTCGCAAATCCCTGT
ACGATCTGACCAAATCCCTGGTGGCGACCTCTCAAGTGGAAGACCTGGTGGTGAACCTGGTGCCGCTGGGCCGCGGTGGCGCTAGCG
AAAGCGGCGCCGAGAACCAGTCCGACCGTTTACAGGGGCGTCAACAAGAGCAG**TAA**

V2-1/loop (loop-incorporated Ah2-1 peptide)

ATGAGCAAAACCATTGTTCTGAGCGTGGGTGAAGCGACCCGTACCCTGACCGAAATCCAGAGCACCGCTGACCGTCAAATTTTTGAGG
AAAAAGTGGGTCCGCTGGTTGGCCGTCTGCGTCTGACCGCGAGCCTGCGTCAGAACGGTGCGAAGACCGCGTACCGTGTGAACCTGA
AACTGGACCAAGCGGATGTGGTTGATTGCAGCACCAGCGTTTGCGGCGAGCTGCCGAAAGTGCGTTACACCCAGGTTTGGAGCCACG
ATGTGACCATCGTTGCGAACAGCACCGAAGCGAGCCGTAAGAGCCTGTATGATCTGACCAAAAGCCTGGTGGCGACCAGCCAAGTTGA
GGACCTGGTGGTTAATCTTGTACCACTTGGTCGCGCATATGGCGATCCGTATAGCCCATCTCAGGACCCATACTCGCCGAGCCCTTAC
GACCGTCGCGGCGCTGGTTCGTCCCAAGGTTCGAAAACCATCGTCCTGTCCGTGGGCGAAGCAACCCGCACCCTGACCGAAATCCAA
TCTACCGCAGACCGCCAAATCTTTGAAGAAAAAGTGGGTCCGCTGGTCGGTCGTCTGCGTCTGACCGCCTCTCTGCGTCAGAACGGCG
CGAAAACGGCCTATCGCGTCAATCTGAAACTGGATCAAGCAGACGTGGTTGATTGCAGCACCTCTGTTTGTGGTGAACTGCCGAAAGTG
CGTTATACGCAGGTTTGGTCACATGACGTCACCATTGTGGCAAACTCGACGGAAGCTAGTCGCAAATCCCTGTACGATCTGACCAAATC
CCTGGTGGCGACCTCTCAAGTGGAAGACCTGGTGGTGAACCTGGTGCCGCTGGGCCGC**TAA**

V2-2b/loop (loop-incorporated Ah2-2b peptide)

ATGAGCAAAACCATTGTTCTGAGCGTGGGTGAAGCGACCCGTACCCTGACCGAAATCCAGAGCACCGCTGACCGTCAAATTTTTGAGG
AAAAAGTGGGTCCGCTGGTTGGCCGTCTGCGTCTGACCGCGAGCCTGCGTCAGAACGGTGCGAAGACCGCGTACCGTGTGAACCTGA
AACTGGACCAAGCGGATGTGGTTGATTGCAGCACCAGCGTTTGCGGCGAGCTGCCGAAAGTGCGTTACACCCAGGTTTGGAGCCACG
ATGTGACCATCGTTGCGAACAGCACCGAAGCGAGCCGTAAGAGCCTGTATGATCTGACCAAAAGCCTGGTGGCGACCAGCCAAGTTGA
GGACCTGGTGGTTAATCTTGTACCACTTGGTCGCGCATATGGCGGGTTGCGTGCGCCTCAACGTTGTGACCTTGACGTAGAAAGTGGA
GGTCGTGATCGTTACGGTTCGAAAACCATCGTCCTGTCCGTGGGCGAAGCAACCCGCACCCTGACCGAAATCCAATCTACCGCAGACC
GCCAAATCTTTGAAGAAAAAGTGGGTCCGCTGGTCGGTCGTCTGCGTCTGACCGCCTCTCTGCGTCAGAACGGCGCGAAAACGGCCTA
TCGCGTCAATCTGAAACTGGATCAAGCAGACGTGGTTGATTGCAGCACCTCTGTTTGTGGTGAACTGCCGAAAGTGCGTTATACGCAGG
TTTGGTCACATGACGTCACCATTGTGGCAAACTCGACGGAAGCTAGTCGCAAATCCCTGTACGATCTGACCAAATCCCTGGTGGCGACC
TCTCAAGTGGAAGACCTGGTGGTGAACCTGGTGCCGCTGGGCCGC**TAA**

V2-6/loop (loop-incorporated Ah2-6 peptide)

ATGAGCAAAACCATTGTTCTGAGCGTGGGTGAAGCGACCCGTACCCTGACCGAAATCCAGAGCACCGCTGACCGTCAAATTTTTGAGG
AAAAAGTGGGTCCGCTGGTTGGCCGTCTGCGTCTGACCGCGAGCCTGCGTCAGAACGGTGCGAAGACCGCGTACCGTGTGAACCTGA
AACTGGACCAAGCGGATGTGGTTGATTGCAGCACCAGCGTTTGCGGCGAGCTGCCGAAAGTGCGTTACACCCAGGTTTGGAGCCACG
ATGTGACCATCGTTGCGAACAGCACCGAAGCGAGCCGTAAGAGCCTGTATGATCTGACCAAAAGCCTGGTGGCGACCAGCCAAGTTGA
GGACCTGGTGGTTAATCTTGTACCACTTGGTCGCGCATATGGCCACGCATCAGCTCGTCAACAGTGGGAGCTTCAGGGTGATCGTCGC
TGTCAAAGTCAGTTGGAGCGTGCGAATGGTTCGAAAACCATCGTCCTGTCCGTGGGCGAAGCAACCCGCACCCTGACCGAAATCCAAT
CTACCGCAGACCGCCAAATCTTTGAAGAAAAAGTGGGTCCGCTGGTCGGTCGTCTGCGTCTGACCGCCTCTCTGCGTCAGAACGGCGC
GAAAACGGCCTATCGCGTCAATCTGAAACTGGATCAAGCAGACGTGGTTGATTGCAGCACCTCTGTTTGTGGTGAACTGCCGAAAGTGC
GTTATACGCAGGTTTGGTCACATGACGTCACCATTGTGGCAAACTCGACGGAAGCTAGTCGCAAATCCCTGTACGATCTGACCAAATCC
CTGGTGGCGACCTCTCAAGTGGAAGACCTGGTGGTGAACCTGGTGCCGCTGGGCCGC**TAA**

V2-7/loop (loop-incorporated Ah2-7 peptide)

ATGAGCAAAACCATTGTTCTGAGCGTGGGTGAAGCGACCCGTACCCTGACCGAAATCCAGAGCACCGCTGACCGTCAAATTTTTGAGG
AAAAAGTGGGTCCGCTGGTTGGCCGTCTGCGTCTGACCGCGAGCCTGCGTCAGAACGGTGCGAAGACCGCGTACCGTGTGAACCTGA
AACTGGACCAAGCGGATGTGGTTGATTGCAGCACCAGCGTTTGCGGCGAGCTGCCGAAAGTGCGTTACACCCAGGTTTGGAGCCACG
ATGTGACCATCGTTGCGAACAGCACCGAAGCGAGCCGTAAGAGCCTGTATGATCTGACCAAAAGCCTGGTGGCGACCAGCCAAGTTGA
GGACCTGGTGGTTAATCTTGTACCACTTGGTCGCGCATATGGCGGTGATCGTCGTTGCCAGAGCCAACTGGAACGTGCCAATCTTCGC
CCATGCGAACAACATTTAATGCAAAAAGGTTCGAAAACCATCGTCCTGTCCGTGGGCGAAGCAACCCGCACCCTGACCGAAATCCAATC
TACCGCAGACCGCCAAATCTTTGAAGAAAAAGTGGGTCCGCTGGTCGGTCGTCTGCGTCTGACCGCCTCTCTGCGTCAGAACGGCGCG
AAAACGGCCTATCGCGTCAATCTGAAACTGGATCAAGCAGACGTGGTTGATTGCAGCACCTCTGTTTGTGGTGAACTGCCGAAAGTGCG
TTATACGCAGGTTTGGTCACATGACGTCACCATTGTGGCAAACTCGACGGAAGCTAGTCGCAAATCCCTGTACGATCTGACCAAATCCC
TGGTGGCGACCTCTCAAGTGGAAGACCTGGTGGTGAACCTGGTGCCGCTGGGCCGC**TAA**

V2-8/loop (loop-incorporated Ah2-8 peptide)

ATGAGCAAAACCATTGTTCTGAGCGTGGGTGAAGCGACCCGTACCCTGACCGAAATCCAGAGCACCGCTGACCGTCAAATTTTTGAGG
AAAAAGTGGGTCCGCTGGTTGGCCGTCTGCGTCTGACCGCGAGCCTGCGTCAGAACGGTGCGAAGACCGCGTACCGTGTGAACCTGA
AACTGGACCAAGCGGATGTGGTTGATTGCAGCACCAGCGTTTGCGGCGAGCTGCCGAAAGTGCGTTACACCCAGGTTTGGAGCCACG
ATGTGACCATCGTTGCGAACAGCACCGAAGCGAGCCGTAAGAGCCTGTATGATCTGACCAAAAGCCTGGTGGCGACCAGCCAAGTTGA
GGACCTGGTGGTTAATCTTGTACCACTTGGTCGCGCATATGGCAACTTACGTCCGTGTGAACAGCATCTTATGCAAAAGATCCAGCGCG
ACGAAGACTCCTATGAACGCGACCCATACTCGGGTTCGAAAACCATCGTCCTGTCCGTGGGCGAAGCAACCCGCACCCTGACCGAAAT
CCAATCTACCGCAGACCGCCAAATCTTTGAAGAAAAAGTGGGTCCGCTGGTCGGTCGTCTGCGTCTGACCGCCTCTCTGCGTCAGAAC
GGCGCGAAAACGGCCTATCGCGTCAATCTGAAACTGGATCAAGCAGACGTGGTTGATTGCAGCACCTCTGTTTGTGGTGAACTGCCGA
AAGTGCGTTATACGCAGGTTTGGTCACATGACGTCACCATTGTGGCAAACTCGACGGAAGCTAGTCGCAAATCCCTGTACGATCTGACC
AAATCCCTGGTGGCGACCTCTCAAGTGGAAGACCTGGTGGTGAACCTGGTGCCGCTGGGCCGC**TAA**

V2-9/loop (loop-incorporated Ah2-9 peptide)

ATGAGCAAAACCATTGTTCTGAGCGTGGGTGAAGCGACCCGTACCCTGACCGAAATCCAGAGCACCGCTGACCGTCAAATTTTTGAGG
AAAAAGTGGGTCCGCTGGTTGGCCGTCTGCGTCTGACCGCGAGCCTGCGTCAGAACGGTGCGAAGACCGCGTACCGTGTGAACCTGA
AACTGGACCAAGCGGATGTGGTTGATTGCAGCACCAGCGTTTGCGGCGAGCTGCCGAAAGTGCGTTACACCCAGGTTTGGAGCCACG
ATGTGACCATCGTTGCGAACAGCACCGAAGCGAGCCGTAAGAGCCTGTATGATCTGACCAAAAGCCTGGTGGCGACCAGCCAAGTTGA
GGACCTGGTGGTTAATCTTGTACCACTTGGTCGCGCATATGGCGAGAACCAGTCCGACCGTTTACAGGGGCGTCAACAAGAGCAGGGT
TCGAAAACCATCGTCCTGTCCGTGGGCGAAGCAACCCGCACCCTGACCGAAATCCAATCTACCGCAGACCGCCAAATCTTTGAAGAAA
AAGTGGGTCCGCTGGTCGGTCGTCTGCGTCTGACCGCCTCTCTGCGTCAGAACGGCGCGAAAACGGCCTATCGCGTCAATCTGAAACT
GGATCAAGCAGACGTGGTTGATTGCAGCACCTCTGTTTGTGGTGAACTGCCGAAAGTGCGTTATACGCAGGTTTGGTCACATGACGTCA
CCATTGTGGCAAACTCGACGGAAGCTAGTCGCAAATCCCTGTACGATCTGACCAAATCCCTGGTGGCGACCTCTCAAGTGGAAGACCT
GGTGGTGAACCTGGTGCCGCTGGGCCGC**TAA**

b. Encapsulin-based peanut epitope nanoparticles

MxEncA gene
Luminally-oriented His$_{6x}$ tag
C-terminal 9 amino acid spacer
Ara h 2 peptide sequence
Start and stop codons are underlined and **bolded**, respectively.

E2-1/loop (loop-incorporated Ah2-1 peptide)

ATGGGCAGCAGCCATCATCATCATCATCACAGCAGCGGCCTGGTGCCGCGCGGCAGCCATATGCCGGATTTCCTCGGTCATGCGGAA
AATCCGCTGCGTGAGGAAGAATGGGCCCGTCTTAACGAAACGGTCATTCAGGTCGCTCGTCGCAGTCTGGTTGGTCGTCGGATTCTGG
ACATTTACGGGCCATTAGGTGCCGGAGTACAAACCGTCCCGTATGACGAGTTTCAGGGCGATCCGTATAGCCCATCTCAGGACCCATA
CTCGCCGAGCCCTTACGACCGTCGCGGCGCTGGTTCGTCCCAAGTTAGCCCTGGTGCTGTCGATATTGTGGGTGAACAGGAAACTGC
GATGGTGTTTACGGATGCCCGTAAATTCAAGACCATTCCCATCATCTACAAAGACTTCCTGCTGCATTGGCGGGATATCGAAGCAGCAC
GCACACACAACATGCCGTTAGACGTATCGGCAGCAGCAGGGGCCGCTGCGCTTTGTGCTCAACAGGAGGACGAACTGATTTTCTATGG
CGATGCGCGTTTGGGGTATGAAGGCCTGATGACCGCCAATGGTCGCCTGACCGTGCCTCTGGGAGATTGGACCAGTCCGGGTGGCGG
CTTTCAGGCGATTGTGGAAGCCACGCGCAAACTGAATGAGCAAGGCCACTTTGGTCCGTATGCGGTTGTGTTATCGCCACGCCTGTAC
TCCCAATTGCATCGCATCTACGAGAAAACGGGCGTTCTGGAGATTGAAACCATTCGCCAGTTAGCATCAGATGGCGTGTATCAGAGCAA
TCGTCTGCGTGGCGAATCTGGCGTCGTGGTATCTACAGGGCGCGAGAACATGGATCTTGCCGTTTCTATGGACATGGTTGCGGCTTAT
CTGGGCGCGAGCCGCATGAACCATCCGTTTCGCGTGTTGGAAGCGCTCTTGCTCCGCATCAAACACCCCGATGCGATCTGCACTCTGG
AAGGAGCCGGTGCGACCGAACGCCGT**TAA**

E2-1/lin (C-terminal Ah2-1 peptide)

ATGGGCAGCAGCCATCATCATCATCATCACAGCAGCGGCCTGGTGCCGCGCGGCAGCCATATGCCGGATTTCCTCGGTCATGCGGAA
AATCCGCTGCGTGAGGAAGAATGGGCCCGTCTTAACGAAACGGTCATTCAGGTCGCTCGTCGCAGTCTGGTTGGTCGTCGGATTCTGG
ACATTTACGGGCCATTAGGTGCCGGAGTACAAACCGTCCCGTATGACGAGTTTCAGGGCGTTAGCCCTGGTGCTGTCGATATTGTGGG
TGAACAGGAAACTGCGATGGTGTTTACGGATGCCCGTAAATTCAAGACCATTCCCATCATCTACAAAGACTTCCTGCTGCATTGGCGGG
ATATCGAAGCAGCACGCACACACAACATGCCGTTAGACGTATCGGCAGCAGCAGGGGCCGCTGCGCTTTGTGCTCAACAGGAGGACG
AACTGATTTTCTATGGCGATGCGCGTTTGGGGTATGAAGGCCTGATGACCGCCAATGGTCGCCTGACCGTGCCTCTGGGAGATTGGAC
CAGTCCGGGTGGCGGCTTTCAGGCGATTGTGGAAGCCACGCGCAAACTGAATGAGCAAGGCCACTTTGGTCCGTATGCGGTTGTGTTA
TCGCCACGCCTGTACTCCCAATTGCATCGCATCTACGAGAAAACGGGCGTTCTGGAGATTGAAACCATTCGCCAGTTAGCATCAGATGG
CGTGTATCAGAGCAATCGTCTGCGTGGCGAATCTGGCGTCGTGGTATCTACAGGGCGCGAGAACATGGATCTTGCCGTTTCTATGGAC
ATGGTTGCGGCTTATCTGGGCGCGAGCCGCATGAACCATCCGTTTCGCGTGTTGGAAGCGCTCTTGCTCCGCATCAAACACCCCGATG
CGATCTGCACTCTGGAAGGAGCCGGTGCGACCGAACGCCGTTCAGGTGGCGCTAGCGAAAGTGGTGCAGATCCGTATAGCCCATCTC
AGGACCCATACTCGCCGAGCCCTTACGACCGTCGCGGCGCTGGTTCGTCCCAA**TAA**

E2-6/loop (loop-incorporated Ah2-6 peptide)

ATGGGCAGCAGCCATCATCATCATCATCACAGCAGCGGCCTGGTGCCGCGCGGCAGCCATATGCCGGATTTCCTCGGTCATGCGGAA
AATCCGCTGCGTGAGGAAGAATGGGCCCGTCTTAACGAAACGGTCATTCAGGTCGCTCGTCGCAGTCTGGTTGGTCGTCGGATTCTGG
ACATTTACGGGCCATTAGGTGCCGGAGTACAAACCGTCCCGTATGACGAGTTTCAGGGCCACGCATCAGCTCGTCAACAGTGGGAGCT
TCAGGGTGATCGTCGCTGTCAAAGTCAGTTGGAGCGTGCGAATGTTAGCCCTGGTGCTGTCGATATTGTGGGTGAACAGGAAACTGCG
ATGGTGTTTACGGATGCCCGTAAATTCAAGACCATTCCCATCATCTACAAAGACTTCCTGCTGCATTGGCGGGATATCGAAGCAGCACG
CACACACAACATGCCGTTAGACGTATCGGCAGCAGCAGGGGCCGCTGCGCTTTGTGCTCAACAGGAGGACGAACTGATTTTCTATGGC
GATGCGCGTTTGGGGTATGAAGGCCTGATGACCGCCAATGGTCGCCTGACCGTGCCTCTGGGAGATTGGACCAGTCCGGGTGGCGGC
TTTCAGGCGATTGTGGAAGCCACGCGCAAACTGAATGAGCAAGGCCACTTTGGTCCGTATGCGGTTGTGTTATCGCCACGCCTGTACT
CCCAATTGCATCGCATCTACGAGAAAACGGGCGTTCTGGAGATTGAAACCATTCGCCAGTTAGCATCAGATGGCGTGTATCAGAGCAAT
CGTCTGCGTGGCGAATCTGGCGTCGTGGTATCTACAGGGCGCGAGAACATGGATCTTGCCGTTTCTATGGACATGGTTGCGGCTTATC
TGGGCGCGAGCCGCATGAACCATCCGTTTCGCGTGTTGGAAGCGCTCTTGCTCCGCATCAAACACCCCGATGCGATCTGCACTCTGGA
AGGAGCCGGTGCGACCGAACGCCGT**TAA**

E2-6/lin (C-terminal Ah2-6 peptide)

ATGGGCAGCAGCCATCATCATCATCATCACAGCAGCGGCCTGGTGCCGCGCGGCAGCCATATGCCGGATTTCCTCGGTCATGCGGAA
AATCCGCTGCGTGAGGAAGAATGGGCCCGTCTTAACGAAACGGTCATTCAGGTCGCTCGTCGCAGTCTGGTTGGTCGTCGGATTCTGG
ACATTTACGGGCCATTAGGTGCCGGAGTACAAACCGTCCCGTATGACGAGTTTCAGGGCGTTAGCCCTGGTGCTGTCGATATTGTGGG
TGAACAGGAAACTGCGATGGTGTTTACGGATGCCCGTAAATTCAAGACCATTCCCATCATCTACAAAGACTTCCTGCTGCATTGGCGGG
ATATCGAAGCAGCACGCACACACAACATGCCGTTAGACGTATCGGCAGCAGCAGGGGCCGCTGCGCTTTGTGCTCAACAGGAGGACG
AACTGATTTTCTATGGCGATGCGCGTTTGGGGTATGAAGGCCTGATGACCGCCAATGGTCGCCTGACCGTGCCTCTGGGAGATTGGAC
CAGTCCGGGTGGCGGCTTTCAGGCGATTGTGGAAGCCACGCGCAAACTGAATGAGCAAGGCCACTTTGGTCCGTATGCGGTTGTGTTA
TCGCCACGCCTGTACTCCCAATTGCATCGCATCTACGAGAAAACGGGCGTTCTGGAGATTGAAACCATTCGCCAGTTAGCATCAGATGG
CGTGTATCAGAGCAATCGTCTGCGTGGCGAATCTGGCGTCGTGGTATCTACAGGGCGCGAGAACATGGATCTTGCCGTTTCTATGGAC
ATGGTTGCGGCTTATCTGGGCGCGAGCCGCATGAACCATCCGTTTCGCGTGTTGGAAGCGCTCTTGCTCCGCATCAAACACCCCGATG
CGATCTGCACTCTGGAAGGAGCCGGTGCGACCGAACGCCGTTCAGGTGGCGCTAGCGAAAGTGGTGCACACGCATCAGCTCGTCAAC
AGTGGGAGCTTCAGGGTGATCGTCGCTGTCAAAGTCAGTTGGAGCGTGCGAAT**TAA**

c. Sequences of nanoparticles which did not express in *E. coli*

PP7-PP7 with C-terminal Ah2-4 peptide

ATGAGCAAAACCATTGTTCTGAGCGTGGGTGAAGCGACCCGTACCCTGACCGAAATCCAGAGCACCGCTGACCGTCAAATTTTTGAGG
AAAAAGTGGGTCCGCTGGTTGGCCGTCTGCGTCTGACCGCGAGCCTGCGTCAGAACGGTGCGAAGACCGCGTACCGTGTGAACCTGA
AACTGGACCAAGCGGATGTGGTTGATTGCAGCACCAGCGTTTGCGGCGAGCTGCCGAAAGTGCGTTACACCCAGGTTTGGAGCCACG
ATGTGACCATCGTTGCGAACAGCACCGAAGCGAGCCGTAAGAGCCTGTATGATCTGACCAAAAGCCTGGTGGCGACCAGCCAAGTTGA
GGACCTGGTGGTTAATCTTGTACCACTTGGTCGCGCATATGGCGGTTCGAAAACCATCGTCCTGTCCGTGGGCGAAGCAACCCGCACC
CTGACCGAAATCCAATCTACCGCAGACCGCCAAATCTTTGAAGAAAAAGTGGGTCCGCTGGTCGGTCGTCTGCGTCTGACCGCCTCTC
TGCGTCAGAACGGCGCGAAAACGGCCTATCGCGTCAATCTGAAACTGGATCAAGCAGACGTGGTTGATTGCAGCACCTCTGTTTGTGG
TGAACTGCCGAAAGTGCGTTATACGCAGGTTTGGTCACATGACGTCACCATTGTGGCAAACTCGACGGAAGCTAGTCGCAAATCCCTGT
ACGATCTGACCAAATCCCTGGTGGCGACCTCTCAAGTGGAAGACCTGGTGGTGAACCTGGTGCCGCTGGGCCGCGGTGGCGCTAGCG
AAAGCGGCGCCCGTGCAAATTTGCGCCCCTGCGAACAACATCTTATGCAAAAAATCCAACGCGACGAGGACTCGTATGAACGTGACCC
TTACTCTCCCTCCCAGGACCCGTACTCTCCGAGCCCATATGATCGTCGC**TAA**

PP7-PP7 with loop-incorporated Ah2-4 peptide

ATGAGCAAAACCATTGTTCTGAGCGTGGGTGAAGCGACCCGTACCCTGACCGAAATCCAGAGCACCGCTGACCGTCAAATTTTTGAGG
AAAAAGTGGGTCCGCTGGTTGGCCGTCTGCGTCTGACCGCGAGCCTGCGTCAGAACGGTGCGAAGACCGCGTACCGTGTGAACCTGA
AACTGGACCAAGCGGATGTGGTTGATTGCAGCACCAGCGTTTGCGGCGAGCTGCCGAAAGTGCGTTACACCCAGGTTTGGAGCCACG
ATGTGACCATCGTTGCGAACAGCACCGAAGCGAGCCGTAAGAGCCTGTATGATCTGACCAAAAGCCTGGTGGCGACCAGCCAAGTTGA
GGACCTGGTGGTTAATCTTGTACCACTTGGTCGCGCATATGGCCGTGCAAATTTGCGCCCCTGCGAACAACATCTTATGCAAAAAATCC
AACGCGACGAGGACTCGTATGAACGTGACCCTTACTCTCCCTCCCAGGACCCGTACTCTCCGAGCCCATATGATCGTCGCGGTTCGAA
AACCATCGTCCTGTCCGTGGGCGAAGCAACCCGCACCCTGACCGAAATCCAATCTACCGCAGACCGCCAAATCTTTGAAGAAAAAGTG
GGTCCGCTGGTCGGTCGTCTGCGTCTGACCGCCTCTCTGCGTCAGAACGGCGCGAAAACGGCCTATCGCGTCAATCTGAAACTGGATC
AAGCAGACGTGGTTGATTGCAGCACCTCTGTTTGTGGTGAACTGCCGAAAGTGCGTTATACGCAGGTTTGGTCACATGACGTCACCATT
GTGGCAAACTCGACGGAAGCTAGTCGCAAATCCCTGTACGATCTGACCAAATCCCTGGTGGCGACCTCTCAAGTGGAAGACCTGGTGG
TGAACCTGGTGCCGCTGGGCCGC**TAA**

PP7-PP7 with C-terminal Ah2-5 peptide

ATGAGCAAAACCATTGTTCTGAGCGTGGGTGAAGCGACCCGTACCCTGACCGAAATCCAGAGCACCGCTGACCGTCAAATTTTTGAGG
AAAAAGTGGGTCCGCTGGTTGGCCGTCTGCGTCTGACCGCGAGCCTGCGTCAGAACGGTGCGAAGACCGCGTACCGTGTGAACCTGA
AACTGGACCAAGCGGATGTGGTTGATTGCAGCACCAGCGTTTGCGGCGAGCTGCCGAAAGTGCGTTACACCCAGGTTTGGAGCCACG
ATGTGACCATCGTTGCGAACAGCACCGAAGCGAGCCGTAAGAGCCTGTATGATCTGACCAAAAGCCTGGTGGCGACCAGCCAAGTTGA
GGACCTGGTGGTTAATCTTGTACCACTTGGTCGCGCATATGGCGGTTCGAAAACCATCGTCCTGTCCGTGGGCGAAGCAACCCGCACC
CTGACCGAAATCCAATCTACCGCAGACCGCCAAATCTTTGAAGAAAAAGTGGGTCCGCTGGTCGGTCGTCTGCGTCTGACCGCCTCTC
TGCGTCAGAACGGCGCGAAAACGGCCTATCGCGTCAATCTGAAACTGGATCAAGCAGACGTGGTTGATTGCAGCACCTCTGTTTGTGG
TGAACTGCCGAAAGTGCGTTATACGCAGGTTTGGTCACATGACGTCACCATTGTGGCAAACTCGACGGAAGCTAGTCGCAAATCCCTGT
ACGATCTGACCAAATCCCTGGTGGCGACCTCTCAAGTGGAAGACCTGGTGGTGAACCTGGTGCCGCTGGGCCGCGGTGGCGCTAGCG
AAAGCGGCGCCCGTTGCTGTAATGAATTGAACGAGTTCGAAAATAATCAGCGTTGCATGTGCGAGGCTTTACAACAGATTATGGAAAAT
CAATCGGATCGCTTGCAGGGTCGCCAGCAAGAGCAA**TAA**

II. Primer sequences for peanut epitope nanoparticle production

Primer name	Primer sequence (5' → 3')
PP7-PP7_Ah2-1_loop_for	AGCCTTTACGACCGTCGCGGCGCTGGTTCGTCCCAAGGTTCGAAAACAATTGTCCTGTC
PP7-PP7_Ah2-1_loop_rev	GCCCGAGTATGGGTCCTGAGATGGGCTATACGGATCGCCATATGCGCGACCAAG
PP7-PP7_Ah2-1_Cterm_for	CTTACGACCGTCGCGGCGCTGGTTCGTCCCAATGATGATAACAATTGCGAGCACCACC
PP7-PP7_Ah2-1_Cterm_rev	GGCTCGGCGAGTATGGGTCCTGAGATGGGCTATACGGATCGGCGCCGCTTTCGCTAGC
PP7-PP7_Ah2-2a_Cterm_for	GACGTAGAGAGCGGGGGGCGCGACCGCTACTGATGATAACAATTGCGAGCACCACC
PP7-PP7_Ah2-2a_Cterm_rev	AAGGTCACAACGTTGAGGCGCACGCAACCCGGCGCCGCTTTCGCTAGC
PP7-PP7_Ah2-2b_Cterm_for	GACGTAGAGAGCGGGGGGCGCGACCGCTACTGATGATAACAATTGCGAGCACCACC
PP7-PP7_Ah2-2b_Cterm_rev	AAGGTCTGAACGTTGAGGCGCACGCAACCCGGCGCCGCTTTCGCTAGC
PP7-PP7_Ah2-2b_loop_for	CGTAGAAAGTGGAGGTCGTGATCGTTACGGTTCGAAAACAATTGTCCTG
PP7-PP7_Ah2-2b_loop_rev	TCAAGGTCACAACGTTGAGGCGCACGCAACCCGCCATATGCGCGACCAAG
PP7-PP7_Ah2-3_Cterm_for	TTAGAGCGTGCGAATTTGCGCCCCTGCGAGCAGCATCTTATGCAGAAGATTCAGCGTTGATGA TAACAATTGCGAGCACCACC
PP7-PP7_Ah2-3_Cterm_rev	CTGTGACTGACAACGGCGGTCACCTTGTAATTCCCATTGCTGGCGCGCCGACGCATGGGCTG CCAGGGCGCCGCTTTCGCTAGC
PP7-PP7_Ah2-4_loop_for	GAACGTGACCCTTACTCTCCCTCCCAGGACCCGTACTCTCCGAGCCCATATGATCGTCGCGGT TCGAAAACAATTGTCCTGTC
PP7-PP7_Ah2-4_loop_rev	ATACGAGTCCTCGTCGCGTTGGATTTTTTGCATAAGATGTTGTTCGCAGGGGCGCAAATTTGC ACGGCCATATGCGCGACCAAG
PP7-PP7_Ah2-4_Cterm_for	AACGTGACCCTTACTCTCCCTCCCAGGACCCGTACTCTCCGAGCCCATATGATCGTCGCTGAT GATAACAATTGCGAGCACCACC
PP7-PP7_Ah2-4_Cterm_rev	CATACGAGTCCTCGTCGCGTTGGATTTTTTGCATAAGATGTTGTTCGCAGGGGCGCAAATTTG CACGGGCGCCGCTTTCGCTAGC
PP7-PP7_Ah2-5_Cterm_for	AACAGATTATGGAAAATCAATCGGATCGCTTGCAGGGTCGCCAGCAAGAGCAATGATGATAAC AATTGCGAGCACCACC
PP7-PP7_Ah2-5_Cterm_rev	GTAAAGCCTCGCACATGCAACGCTGATTATTTTCGAACTCGTTCAATTCATTACAGCAACGGGC GCCGCTTTCGCTAGC
PP7-PP7_Ah2-6_loop_for	TCGTCGCTGTCAAAGTCAGTTGGAGCGTGCGAATGGTTCGAAAACAATTGTCCTG
PP7-PP7_Ah2-6_loop_rev	TCACCCTGAAGCTCCCACTGTTGACGAGCTGATGCGTGGCCATATGCGCGACCAAG
PP7-PP7_Ah2-6_Cterm_for	CGTCGCTGTCAAAGTCAGTTGGAGCGTGCGAATTGATGATAACAATTGCGAGCACC
PP7-PP7_Ah2-6_Cterm_rev	ATCACCCTGAAGCTCCCACTGTTGACGAGCTGATGCGTGGGCGCCGCTTTCGCTAGC
PP7-PP7_Ah2-7_loop_for	TCTTCGCCCATGCGAACAACATTTAATGCAAAAAGGTTCGAAAACAATTGTCCTG
PP7-PP7_Ah2-7_loop_rev	TTGGCACGTTCCAGTTGGCTCTGGCAACGACGATCACCGCCATATGCGCGACCAAG
PP7-PP7_Ah2-7_Cterm_for	TCTTCGCCCATGCGAACAACATTTAATGCAAAAATGATGATAACAATTGCGAGCACC
PP7-PP7_Ah2-7_Cterm_rev	TTGGCACGTTCCAGTTGGCTCTGGCAACGACGATCACCGGCGCCGCTTTCGCTAGC
PP7-PP7_Ah2-8_loop_for	AGCGCGACGAAGACTCCTATGAACGCGACCCATACTCGGGTTCGAAAACAATTGTCCTG
PP7-PP7_Ah2-8_loop_rev	GGATCTTTTGCATAAGATGCTGTTCACACGGACGTAAGTTGCCATATGCGCGACCAAG
PP7-PP7_Ah2-8_Cterm_for	GCGCGACGAAGACTCCTATGAACGCGACCCATACTCGTGATGATAACAATTGCGAGCACC
PP7-PP7_Ah2-8_Cterm_rev	TGGATCTTTTGCATAAGATGCTGTTCACACGGACGTAAGTTGGCGCCGCTTTCGCTAGC
PP7-PP7_Ah2-9_loop_for	GGGCGTCAACAAGAGCAGGGTTCGAAAACAATTGTCCTG
PP7-PP7_Ah2-9_loop_rev	CTGTAAACGGTCGGACTGGTTCTCGCCATATGCGCGACCAAG
PP7-PP7_Ah2-9_Cterm_for	GGGGCGTCAACAAGAGCAGTGATGATAACAATTGCGAGCACC
PP7-PP7_Ah2-9_Cterm_rev	TGTAAACGGTCGGACTGGTTCTCGGCGCCGCTTTCGCTAGC
MxEncA_9aa linker_for	TAAGGATCCGGCTGCTAACAAAGC
MxEncA_9aa linker_rev	TGCACCACTTTCGCTAGCGCCACCTGAACGGCGTTCGGTCGCACC
MxEncA_Ah2-1_loop_for	AGCCCTTACGACCGTCGCGGCGCTGGTTCGTCCCAAGTTAGCCCTGGTGCTGTC
MxEncA_Ah2-1_loop_rev	CGGCGAGTATGGGTCCTGAGATGGGCTATACGGATCGCCCTGAAACTCGTCATACG

MxEncA_Ah2-1_Cterm_for	CTTACGACCGTCGCGGCGCTGGTTCGTCCCAATAAGGATCCTGCTAAGGGTATATTGG
MxEncA_Ah2-1_Cterm_rev	GGCTCGGCGAGTATGGGTCCTGAGATGGGCTATACGGATCTGCACCACTTTCGCTAGC
MxEncA_Ah2-6_loop_for	TGATCGTCGCTGTCAAAGTCAGTTGGAGCGTGCGAATGTTAGCCCTGGTGCTGTC
MxEncA_Ah2-6_loop_rev	CCCTGAAGCTCCCACTGTTGACGAGCTGATGCGTGGCCCTGAAACTCGTCATAC
MxEncA_Ah2-6_Cterm_for	GTCGCTGTCAAAGTCAGTTGGAGCGTGCGAATTAAGGATCCTGCTAAGGGTATATTGG
MxEncA_Ah2-6_Cterm_rev	GATCACCCTGAAGCTCCCACTGTTGACGAGCTGATGCGTGTGCACCACTTTCGCTAGC

III. Materials and Methods

a. Cloning of peanut epitope variants

Peanut peptides were genetically inserted into either dimeric PP7 or *M. xanthus* encapsulin genes via whole-plasmid PCR amplification using mutagenic primers encoding the DNA sequences for the individual peanut peptides. All PCRs were performed using Q5® High-Fidelity DNA Polymerase (New England Biolabs, NEB). Linear double-stranded DNA products were subsequently 5' phosphorylated and ligated into circular plasmids using KLD Enzyme Mix kits (NEB) following the manufacturer's instructions. Recombinant plasmids were propagated in NEB® 5-alpha Competent *E. coli* cells, and all plasmids were verified by Sanger sequencing prior to use. Loop-incorporated peptide display was accomplished using previously established insertion points in the respective nanoparticle (NP) coat protein genes. For loop-incorporated peptide display on PP7 dimer NPs, peanut peptides were inserted between the two glycine residues of the AYGG tetrapeptide linker moiety bridging the two covalently-linked PP7 coat protein copies.[4,5] For loop-incorporated peptide display on encapsulin NPs, peanut peptides were inserted between residues G58 and V59 in the extended loop region of the encapsulin coat protein monomer.[6] For display of linear peptides on NP scaffolds, peanut peptides were appended to the C-termini of both PP7 dimer and encapsulin genes with either an 8- (GGASESGA)[4] or 9-amino acid (SGGASESGA) flexible linker sequence, respectively, bridging the coat protein C-terminus and the N-termini of the peanut peptides.

Encapsulin-based peanut peptide NPs were generated using the previously reported pET14b:*HEnc* encapsulin variant as the template scaffold for whole-plasmid PCR.[6] This variant contains a luminally-oriented N-terminal hexahistidine affinity tag coupled with two copies of an anti-polyhistidine RNA aptamer[7] (encoded in the 5'- and 3'-untranslated regions of the plasmid) to effect RNA packaging within assembled encapsulin NPs during *in vivo* expression. Crucially, such RNA-containing encapsulins have been shown to possess increased immunogenicity relative to unmodified, non-RNA packaging encapsulins.[6] PP7 dimer peanut peptide NPs were produced from one of two plasmid scaffolds: loop-incorporated peptides were cloned into a dimeric PP7 gene in a modified pCDF plasmid backbone while C-terminally extended peptides were appended onto a dimeric PP7 gene inserted into a pET28a plasmid backbone.[4]

b. Overexpression of peanut peptide NPs

All PP7 dimer NPs were produced in BL21(DE3) *E. coli* cells (NEB). NP variants were expressed according to one of three different methods. <u>Method 1</u>: Transformant cells containing a variant plasmid were cultured in 50 mL of 2YT growth medium (supplemented with a final concentration of either 50 ug/mL streptomycin or 50 µg/mL kanamycin for positive selection of the pCDF or pET28a-based vectors, respectively) in a 250 mL baffled Erlenmeyer flask overnight at 37 °C in an orbital shaking incubator. The saturated starter cell culture was subsequently used to seed 500 mL of fresh 2YT medium (supplemented with antibiotic) in a 2 L baffled Erlenmeyer flask at a 1:50 ratio. The 0.5 L expression culture was incubated at 37 °C in an orbital floor incubator until the culture OD_{600} value reached 0.8 to 1.0. Protein expression was then induced by addition of 1 mM IPTG, and the expression

culture was subsequently incubated at 26 °C for 20 hours prior to harvesting the cells by centrifugation. Collected cell pellets were stored at -20 °C prior to use. Method 2: Transformed cells containing a variant plasmid were inoculated directly into 2 L baffled Erlenmeyer flask containing 500 mL of 2YT medium and the appropriate antibiotic. The expression culture was incubated at 26 °C in an orbital floor incubator for 24 hours without IPTG induction. Cells were harvested by centrifugation and stored at -20 °C prior to use. Method 3: For instances in which peanut peptide NPs could not be expressed homogeneously (i.e., with only the peptide-modified PP7 dimer coat protein genes present *in vivo*), hybrid expression was attempted by co-transforming BL21(DE3) *E. coli* cells with both the plasmid containing the peptide-modified PP7 dimer gene and a second plasmid containing an unmodified PP7 dimer gene copy (e.g., co-transformation with a pCDF-based peptide-modified PP7 dimer gene and a pET28a-based plasmid containing an unmodified PP7 dimer gene, or vice versa). Transformant cells were inoculated into 50 mL of 2YT medium containing both streptomycin and kanamycin (final concentration of 50 µg/mL for each antibiotic) in a 250 mL baffled Erlenmeyer flask and were grown to saturation overnight at 37 °C in an orbital floor incubator. Cells from the saturated starter culture were used to seed 500 mL of 2YT medium (supplemented with both streptomycin and kanamycin) in a 2 L baffled Erlenmeyer flask at a 1:50 ratio. All subsequent expression steps were identical to Method 1 detailed above.

All encapsulin-based peanut peptide NPs were also produced in BL21(DE3) *E. coli* cells. Transformant colonies containing variant peanut peptide encapsulin plasmids were inoculated directly into 0.5 L of SOB medium containing a final concentration of 0.1 µg/mL ampicillin and grown to saturation at 26 °C in an orbital floor incubator for 24 hours without IPTG induction. Cells were subsequently harvested by centrifugation and stored at -20 °C prior to use.

c. Purification of peanut peptide NPs

PP7 dimer-based NPs were purified by resuspending the cell pellets from a full 0.5 L expression culture in 50 mL of 50 mM potassium phosphate (pH 7.0) containing a final concentration of 1 mM dithiothreitol (DTT). Resuspended cells were transferred to a 100 mL glass beaker and then lysed via sonication for a total of 10 minutes in an ice water bath using a QSonica Q500 sonicator equipped with ½” probe tip (5 second pulses, 5 seconds rest between pulses, 50-60% amplitude). The resulting cell lysate was clarified by centrifugation at 14,000 rpms in a JA-17 rotor (Beckman Coulter) for 15 minutes at 4 °C. NPs were precipitated out of the clarified lysate by adding solid ammonium sulfate to a final concentration of 0.265 g/mL and incubating the lysate at 4 °C for 1 hour once all solids had dissolved. Precipitated solids were collected by centrifugation in a JA-17 rotor for 15 minutes at 4 °C and were then resuspended in 8 mL of fresh lysis buffer supplemented with 1 mM DTT after decanting the supernatant. Residual hydrophobic contaminants were removed from the resuspended NP sample by organic extraction with an equal volume of a 1:1 mixture of *n*-butanol/chloroform. The aqueous layer was removed by aspiration and then layered on top of 10-40% sucrose gradients (sucrose solutions prepared in 50 mM potassium phosphate [pH 7.0] supplemented with 1 mM DTT). Sucrose gradient sedimentation was then performed via centrifugation at 4 °C for 4 hours at 28,000 rpms in a SW32 rotor (Beckman Coulter). Isolated NPs were removed from the middle of the sucrose gradients by aspiration and were subsequently concentrated by ultracentrifugation at 68,000 rpms for 2 hours at 4 °C in a Type 70Ti rotor (Beckman Coulter). NP pellets were gently resuspended in 1x phosphate buffered saline (PBS) supplemented with 1 mM DTT and were sterile filtered through 0.2 µm PES syringe filters. Purified NPs were stored at -80 °C until use.

Encapsulin-based NPs were purified in a similar fashion to PP7 dimer NPs. Briefly, cell pellets from 0.25 to 0.5 mL of expression culture were resuspended in 50 mL of 50 mM Tris-HCl (pH 8.5) containing final concentrations of 1 mM DTT and 2 µg/mL each of RNase A and DNase I (Millipore Sigma). Resuspended cells were lysed by sonication and the cell lysate was clarified in an identical fashion to the PP7 dimer-based NPs. The clarified cell lysate was then incubated on ice for 30 minutes

before being passed over 2 x 5 mL HiTrap™ Q-FF anion exchange chromatography columns (Cytiva) connected in series (anion exchange columns were pre-equilibrated with 50 mL of lysis buffer). The column flow-through was collected, then an additional 10 mL of lysis buffer was passed over the dual columns and collected in the flow-through sample. Encapsulin NPs were precipitated out of the flow-through sample by adding 6 g PEG-6000 and 1.2 g NaCl to the flow-through sample and incubating the sample at 4 °C for one hour after all the solids had dissolved. Precipitated solids were collected by centrifugation and were then resuspended in 8 mL of 50 mM potassium phosphate buffer (pH 7.0) supplemented with 1 mM DTT. All remaining purification steps were identical to the procedure described for PP7 dimer NPs, including organic extraction, 10-40% sucrose gradient sedimentation, ultracentrifugation, and final resuspension in 1x PBS followed by filtration through a 0.2 μm PES syringe filter. Purified encapsulin NPs were stored at -80°C until use.

d. Characterization of peanut peptide NPs

The protein concentrations of purified peanut peptide NPs were determined via Bradford assays using Coomassie Plus Bradford Assay Kits (Pierce) and bovine serum albumin as the reference protein standard. Dynamic light scattering (DLS) was used to assess NP sample homogeneity. For each DLS run, the NP sample was diluted to a final concentration of 0.1 to 0.15 mg/mL in 1x PBS buffer, and sample data was collected using a Dynapro DLS plate reader (Wyatt Technologies). Individual NPs were visualized by negative stain transmission electron microscopy (TEM) imaging. TEM grids (300 mesh lacey Formvar/carbon grids, Ted Pella Inc.) were prepared by pipetting 8 μL of NP sample (0.05 mg/mL in 1x PBS) onto the grid surface for 90 seconds before blotting the liquid away on a Kimwipe. Residual buffer salts and non-adsorbed proteins were removed by floating the grid sequentially on two 0.5 mL drops of deionized water (10 seconds float time on each water drop). The grid was again blotted dry on a Kimwipe and negative staining was performed by pipetting 8 μL of 2% (w/v) uranyl acetate solution onto the grid surface for 60 seconds. The grid was blotted against a Kipwipe one final time and then air dried at room temperature for 5 minutes. TEM images were collected at 20,000 to 40,000x magnification using a Hitachi HT7700 microscope operating at a 120 kV.

For PP7 dimer NPs requiring hybrid *in vivo* expression with an unmodified PP7 dimer gene copy to form viable NPs, the total number of peanut peptides incorporated into each NP was estimated using Protein80 Bioanalyzer microfluidic denaturing PAGE chips (Agilent Technologies). Integrated peak areas were determined for the unmodified and peptide-added PP7 dimer coat proteins from the microfluidic electropherograms and were used to calculate the average peptide loading per NP with the following formula where PA*m* is the integrated peak area corresponding to peptide-modified PP7 dimer coat proteins, PA*u* is the integrated peak area corresponding to unmodified PP7 dimer coat proteins, and 120 represents the number of coat proteins dimers required to assemble the *T* = 4 icosahedral assembly state characteristic of PP7 dimer VLPs.[2]

$$\text{Avg. peanut peptides per NP} = \left(\frac{\text{PA}m}{\text{PA}m + \text{PA}u}\right) \text{x } 120$$

e. Biotinylated peptides for use in ELISA analyses

N-terminal biotinylated versions of peptides Ah2-1, Ah2-2a, and Ah2-3 were obtained from Genscript with an N-terminal biotin-PEG6 linker. The remaining peptides were synthesized by standard Fmoc-based solid-phase synthesis on Rink amide resin using a CSBio-II automated synthesizer. After completion of the synthesis but before sidechain deprotection and resin cleavage, each peptide was Fmoc-deprotected with 4-methylpiperidine as usual and then treated with biotin-PEG3-NHS ester (BroadPharm catalog number BP-21509). After 15 minutes at room temperature, the reagent was washed away, and deprotection and resin cleavage was performed as usual with a mixture of CF_3CO_2H, water, and ($\underline{i}$Pr)$_3$SiH.

IV. Supplemental Figures

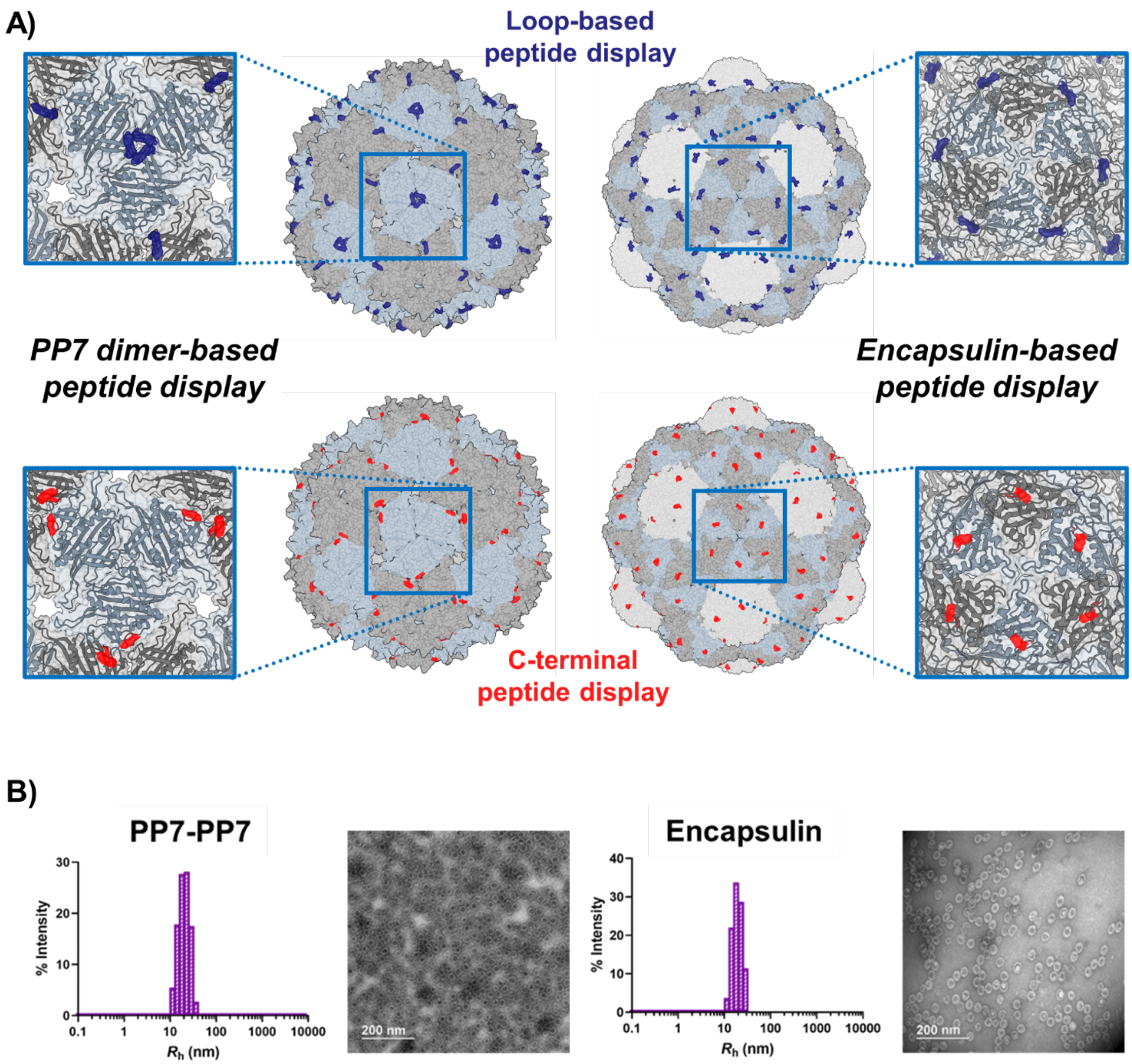

Figure S1. A) Closer structural details of loop and C-terminal peptide insertion points on the PP7 dimer and encapsulin scaffolds chosen for this study. B) DLS and TEM characterization of unmodified PP7 dimer and encapsulin scaffolds.

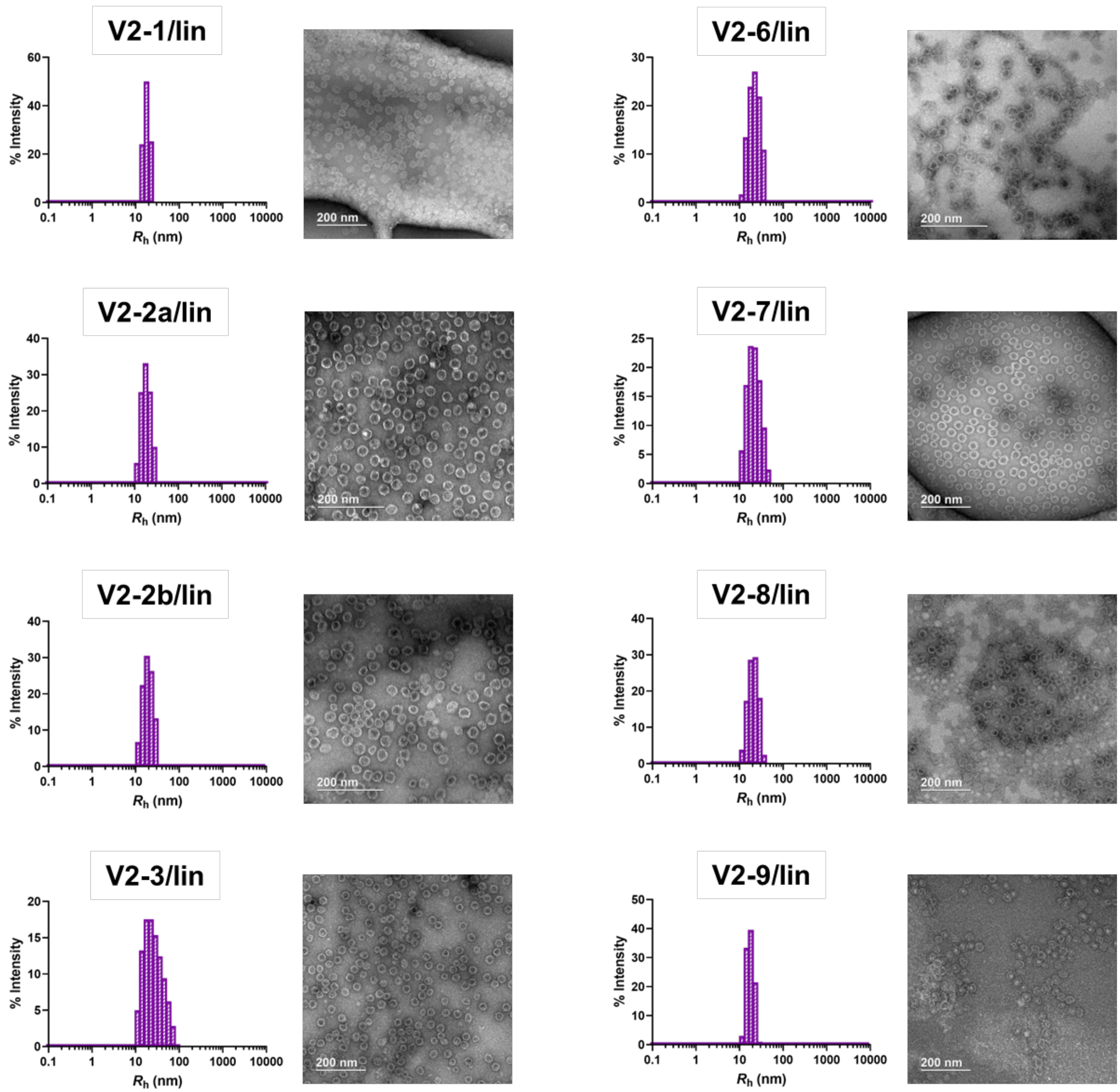

Figure S2. DLS and TEM characterization of PP7 dimer-based NPs presenting peanut peptides as linear extensions from the protomer C-terminus. All TEM images were collected at magnifications between 20,000x to 40,000x. Scale bars of 200 nm are presented for each TEM image.

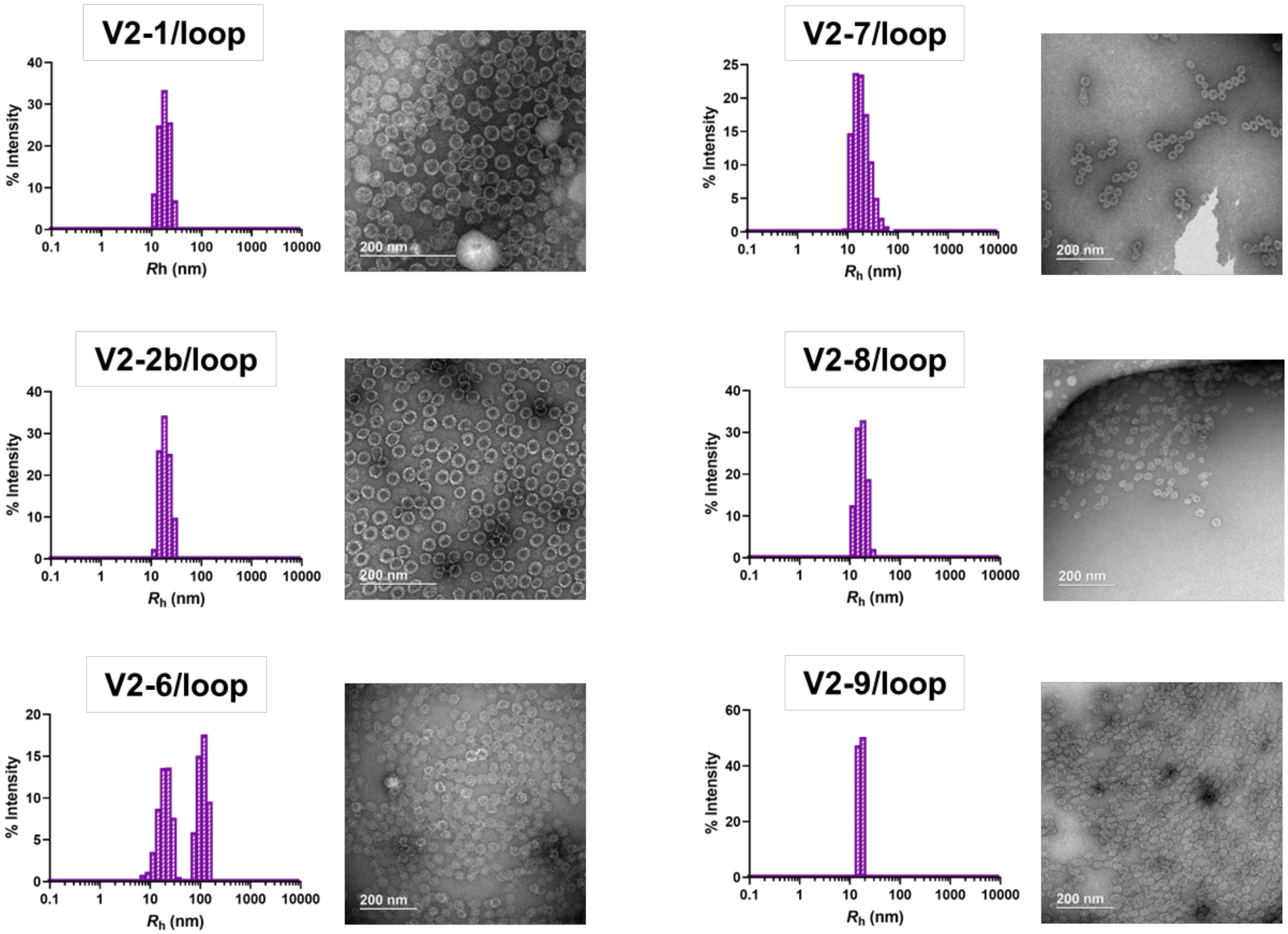

Figure S3. DLS and TEM characterization of PP7 dimer-based NPs presenting peanut peptides as grafts within the AYGG tetrapeptide linker joining the two coat protein dimers. All TEM images were collected at magnifications between 20,000x to 40,000x. Scale bars of 200 nm are presented for each TEM image.

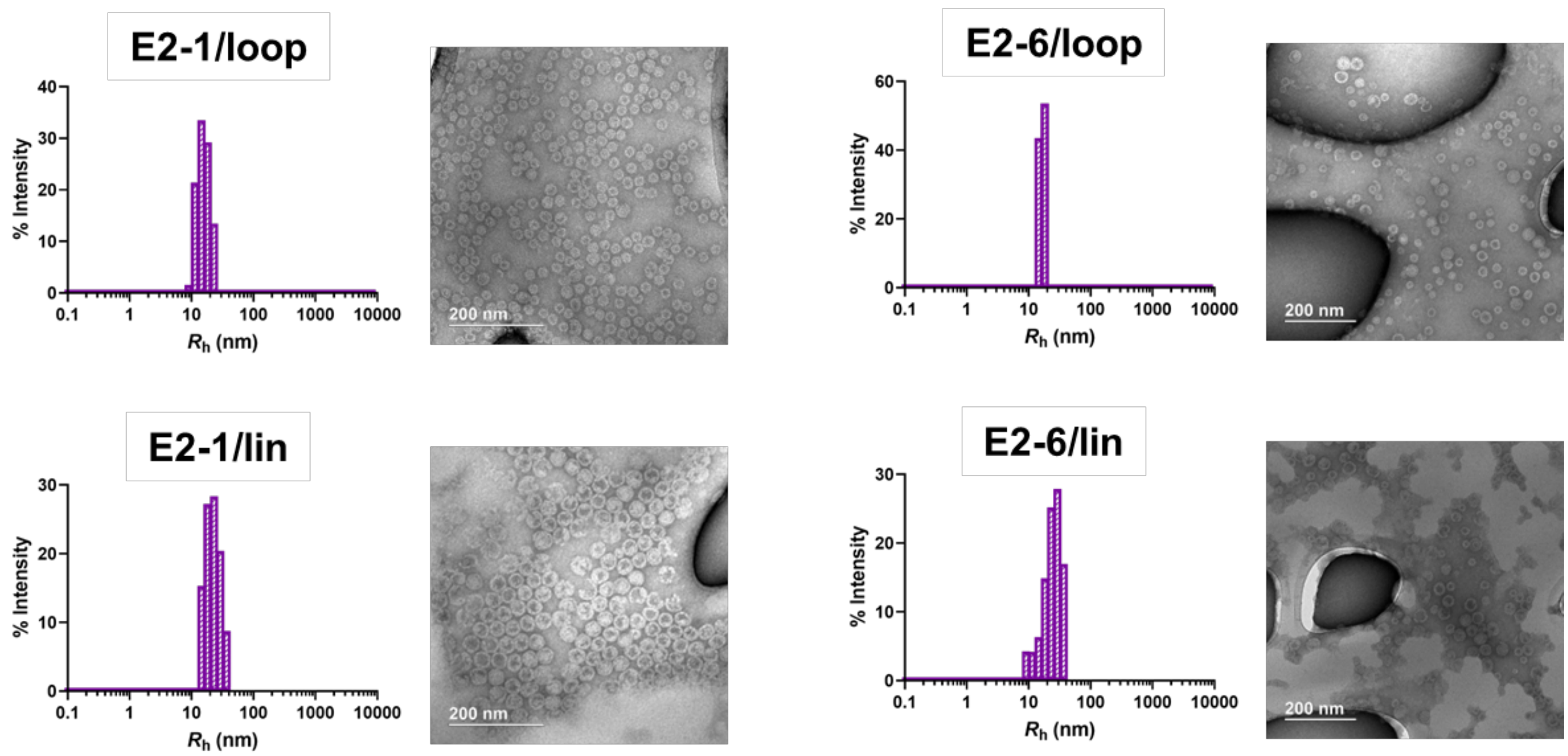

Figure S4. DLS and TEM characterization of *M. xanthus* encapsulin-based NPs. All TEM images were collected at magnifications between 20,000x to 40,000x. Scale bars of 200 nm are presented for each TEM image.

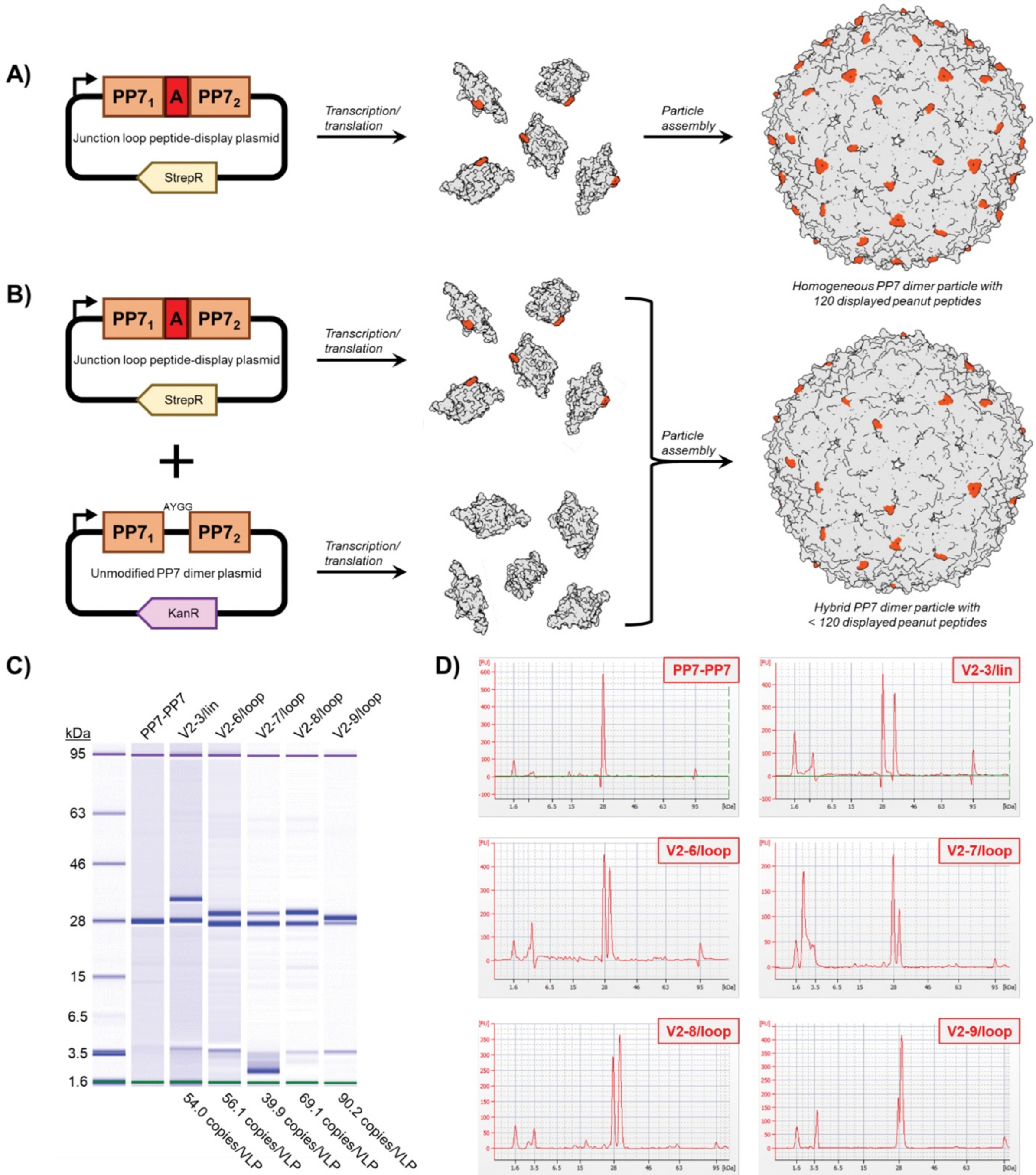

Figure S5. Production and characterization of hybrid-display PP7 dimer nanoparticles. A) Schematic representing the formation of VLPs with 120 surface-presented peptides via homogeneous expression of only modified protomers *in vivo*. B) Schematic depicting the production of reduced valency VLPs from simultaneous coexpression of peptide-modified and wild-type protomers *in vivo*. C) Denaturing microfluidic PAGE image depicting the unmodified PP7 dimer VLPs versus hybrid-expressed PP7 dimer VLPs. D) Electropherograms used to generate the PAGE gel image depicted in C). The number of peanut-peptides per NP was calculated using the integrated peak areas of the unmodified and peptide-added coat protein peaks. Note, individual samples presented in C) were collected on different days using different microfluidic chips. A single representative molecular weight ladder lane was used from the chip on which the PP7-PP7 sample was run for the image in C), thus the protein bands in the subsequent lanes might deviate slightly from the ladder image presented due to slight variances between different microfluidic chips. The molecular weights presented on the x-axes of the electropherograms in D) were generated using the individual molecular weight ladder samples from the specific chips on which each sample was run, and are thus more accurate relative to each sample.

V. References

1. Stanley, J.S., King, N., Burks, A.W., Huang, S.K., Sampson, H., Cockrell, G., Helm, R.M., West, C.M., Bannon, G.A. Identification and Mutational Analysis of the Immunodominant IgE Binding Epitopes of the Major Peanut Allergen Ara h 2. *Arch. Biochem. Biophys.* **1997**, *342*(2), 244-253. https://doi.org/10.1006/abbi.1997.9998
2. Shreffler_W.G., Lencer, D.A., Bardina, L., Sampson, H.A. IgE and IgG4 Epitope Mapping by Microarray Immunoassay Reveals the Diversity of Immune Response to the Peanut Allergen, Ara h 2. *J. Allergy Clin. Immunol*. **2005**, *116*(4) 893-899. https://doi.org/10.1016/j.jaci.2005.06.033
3. Otsu, K., Guo, R., Dreskin, S.C. Epitope Analysis of Ara h 2 and Ara h 6: Characteristic Patterns of IgE-binding Fingerprints Among Individuals with Similar Clinical Histories. *Clin. Exp. Allergy.* **2014**. *45*, 471-484. https://doi.org/10.1111/cea.12407
4. Keshavarz-Joud, P., Zhao, L., Bobe, D., Hernandez, C., Kopylov, M., Yen, L.Y., Djeddar, N., Thompson, B., Connors, C., Gibson, G., Bryksin, A., Finn, M.G. Exploring the Landscape of the PP7 Virus-like Particle for Peptide Display. *ACS Nano* **2023**, *17*(18), 18470-18480. https://doi.org/10.1021/acsnano.3c06178
5. Zhao, L.; Kopylov, M.; Potter, C. S.; Carragher, B.; Finn, M. G. Engineering the PP7 Virus Capsid as a Peptide Display Platform. *ACS Nano* **2019**, *13*, 4443– 4454. https://doi.org/10.1021/acsnano.8b09683
6. Bhattacharya, S., Jenkins, M.C., Keshavarz-Joud, P., Bourque, A.R., White, K., Barkane, A.M.A., Bryksin, A.V., Hernandez, C., Kopylov, M., Finn, M.G. Heterologous Prime-Boost with Immunologically Orthogonal Protein Nanoparticles for Peptide Immunofocusing. *ACS Nano* **2024**, *18*(31), 20083-20100. https://doi.org/10.1021/acsnano.4c00949
7. Tsuji, S.; Tanaka, T.; Hirabayashi, N.; Kato, S.; Akitomi, J.; Egashira, H.; Waga, I.; Ohtsu, T. RNA aptamer binding to polyhistidine-tag. *Biochem. Biophys. Res. Commun.* **2009**, *386*, 227– 231. https://doi.org/10.1016/j.bbrc.2009.06.014
